# Chondrosarcoma: Multi-Targeting Therapeutic Effects of Doxorubicin, BEZ235, and the Small Molecule Aspartyl-Asparaginyl-β-hydroxylase Inhibitor SMI1182

**DOI:** 10.3390/cancers17101671

**Published:** 2025-05-15

**Authors:** Megan Fife, Ming Tong, Bhaskar Das, Rene Rodriguez, Parthiban Chokkalingam, Rolf I. Carlson, Suzanne M. de la Monte

**Affiliations:** 1 Molecular Pharmacology, Physiology, and Biotechnology Graduate Program, Brown University, Providence, RI 02912, USA; 2 Department of Medicine, Rhode Island Hospital, Brown University Health, Providence, RI 02912, USArolf_carlson@brown.edu (R.I.C.); 3 Department of Drug and Biotherapeutic Discovery, School of Pharmacy and Pharmaceutical Sciences, University at Buffalo, State University of New York (SUNY), Buffalo, NY 14201, USA; bhaskard@buffalo.edu (B.D.);; 4 Health Research Institute of Asturias (ISPA), University Institute of Oncology of Asturias (IUOPA), 33001 Oviedo, Spain; rene.rodriguez@ispasturias.es; 5 CIBER Enoncologia (CIBERONC), 28054 Madrid, Spain; 6 Departments of Pathology and Laboratory Medicine, Neurology, and Neurosurgery, Rhode Island Hospital, Women & Infants Hospital, Brown University Health, Alpert Medical School of Brown University, Providence, RI 02912, USA

**Keywords:** aspartyl-asparaginyl-β-hydroxylase, BEZ235, cancer treatment, chondrosarcoma, doxorubicin, Notch, small molecule inhibitor

## Abstract

Background/Objectives: Chondrosarcoma (CS), the most common malignant bone tumor in adults, exhibits a poor prognosis due to high rates of post-surgical recurrence and metastasis, and resistance to chemotherapy. CS’s abundant expression of aspartyl-asparaginyl-β-hydroxylase (ASPH), which drives invasive tumor growth via Notch and PI3K/mTOR activation, opens opportunities for treatment in combination with standard Doxorubicin (DOX) chemotherapy. We hypothesized that the small molecule inhibitor SMI1182, which targets the catalytic domain of ASPH, and BEZ235, which targets PI3K/mTOR, could enhance the chemotherapeutic effects of DOX. Human CS1 (Grade 3) and CDS11 (Grade 2) conventional CS cell lines were treated with broad dose ranges of DOX, BEZ235, or SMI1182 as mono- or combination therapy to assess their anti-tumor effects on cell viability, toxicity, and motility. Methods: Mechanistic studies included the analysis of ASPH expression, Notch signaling, and insulin/IGF/IRS pathway activation through mTOR. DOX, BEZ235, or SMI1182 treatments caused dose-dependent cell loss and cytotoxicity. Results: SMI1182 and BEZ235, with or without DOX, significantly reduced directional motility. Combined treatments had additive cytotoxic effects linked to the reduced expression of ASPH, Notch transcription factors, and insulin receptor substrate type I, which positively regulates both ASPH and Notch. Conclusions: Triple-drug treatment with DOX, SMI1182, and BEZ235 could potentially improve disease-free survival with CS by the simultaneous targeting of multiple upstream mediators of aggressive malignant tumor cell behavior.

## Simple Summary

This study compares molecular features in intermediate and high-grade conventional chondrosarcomas and their responses to individual, combination, and novel chemotherapeutic agents. The findings may account for differences in aggressive tumor behavior in relation to histopathological grade and suggest that chondrosarcoma tumor grade should be factored in for predicting the efficacy of emerging chemotherapeutic approaches.

## 1. Introduction

**Figure 1:**
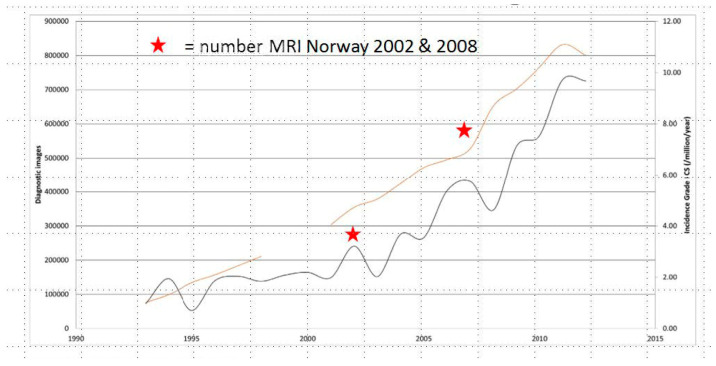
Incidence of ACT/CS grade 1 in the Netherlands between 1989 and 2013, number of MRI examinations over time. Adapted from Thorkildesen and Myklebust (2023) [3]. The gold line corresponds to the number of diagnostic imaging studies performed. The charcoal line reflects the crude incident rates of Grade I CS over time.

Chondrosarcoma (CS) is the most common bone sarcoma in adults [1] and accounts for 20–25% of osseous malignancies. The initial diagnosis of CS is typically made in people 30 to 60 years old, but 80% are older than 40 (https://www.bcrt.org.uk/information/information-by-type/chondrosarcoma/, accessed on 20 April 2025). Among the four main histopathological subtypes, conventional CS accounts for 75%, and de-differentiated, mesenchymal, and clear cell subtypes together account for approximately 25% of CS. In the U.S.A., the crude annual incidence of CS increased over time through 2013 [2], but subsequently, the rates leveled off. In a later national study, Thorkildsen and Myklebust showed that the prevalence rate of CS progressively increased between 2000 and 2020 (Figure 1), although age-adjusted incidence and mortality did not fluctuate significantly over that same interval [3]. Higher prevalence vis-à-vis stable incidence rates may suggest improvements in survival or the detection of treatable recurrent disease.

The standard treatment for CS is wide surgical resection, including the affected bone and surrounding tissue, with wide negative margins [4]. In patients with unresectable chondrosarcoma or metastatic disease, the clinical prognosis is poor [4]. The first-line chemotherapeutic agent for high-grade and advanced sarcomas, including chondrosarcoma, consists of Doxorubicin (DOX) [5,6]. However, high-grade conventional chondrosarcomas are known to be resistant to radiotherapy and chemotherapy [4], and DOX mono-chemotherapy is suboptimum in terms of efficacy, as evidenced by the universally poor outcomes due to pulmonary metastases and mortality [7]. Current standard treatment regimens mainly consist of multimodal combination treatment with 2 or 3 drugs, including DOX plus DNA-damaging agents such as cisplatin or isofamide [4]. Unresectable recurrences and metastasis linked to post-treatment resistance to anticancer agents render chondrosarcoma clinical outcomes discouragingly poor [4,8].

The five-year survival rate with CS is between 26% and 32%, with metastatic disease further reducing mean survival to 23% [9]. Importantly, prognosis is linked to tumor grade such that high-grade CSs exhibit the highest recurrence rates and shortest survivals [9]. Moreover, there is no effective systemic treatment for chondrosarcoma. These statistics, combined with the minimal progress in CS therapeutics, led the American Academy of Orthopaedic Surgeons to prioritize the development of CS treatment strategies. Most likely, newer, more effective therapeutics will require molecular targeting to inhibit CS growth and metastasis. Potential strategies to improve survival with CS include the use of chemotherapeutic agents that target signaling pathways that have roles in chondrosarcoma malignant behavior, such as the PI3K-Akt-mTOR pathway and angiogenesis [10] [11], in addition to genomic mutations of isocitrate dehydrogenase (IDH) IDH1/2, exostosin-1/2 (EXT1/2), COL2A1, TP53, telomerase reverse transcriptase (TERT), and cyclin-dependent kinase inhibitor 2A/B (CDKN2A/B) [4,10,12]. To this end, there are many ongoing but early-stage clinical trials designed to assess the therapeutic efficacy in targeting angiogenesis (PD-L1), mTOR, PI3K, CDK4/6, or IDH1 [4]. In a retrospective study, Molho et al. demonstrated efficacy in treating CS with the mTOR inhibitor sirolimus in combination with cyclophosphamide [8,13].

Another likely important therapeutic target for chondrosarcoma is Notch, which regulates cell proliferation, differentiation, migration, infiltrative growth, and cell fate, and has potential therapeutic efficacy for a broad range of cancers [14,15,16,17,18]. Aberrantly increased Notch activity has been implicated in many malignancies, including chondrosarcoma [19]. The Notch signaling inhibitor LY3039478 was initially reported to be well tolerated as monotherapy for various carcinomas [20], but in a later Phase 1b/II randomized study, although intra-tumoral Notch and downstream signaling through phosphorylated Akt were reduced, there were no significant effects on progression-free or overall survival in a heterogeneous group of sarcomas, including CS [21]. Furthermore, enthusiasm for pursuing Notch-inhibitor chemotherapeutic drugs for advanced disease and forwarding their use in clinical trials has been dampened by the significant off-target effects [17,22,23,24].

Emerging data point toward aspartyl-asparaginyl-β-hydroxylase (ASPH) as an excellent candidate for strategically targeting Notch signaling networks in malignancies. ASPH, a type II transmembrane protein of the α-ketoglutarate-dependent dioxygenase family [25,26], has functional roles in cell motility utilized for infiltrative and metastatic tumor growth [27]. Mechanistically, the C-terminal region of ASPH contains a catalytic site that hydroxylates epidermal growth factor (EGF)-like domains expressed in molecules such as Notch and Jagged [28,29,30,31]. EGF-like domains have important roles in regulating localized signaling of growth and differentiation [32]. ASPH’s interactions with Notch lead to the nuclear translocation of the Notch intracellular domain [33], followed by the activation of transcription factors such as HES and HEY [29,33]. The abolishment of ASPH’s catalytic activity by either site-directed mutagenesis of its critical His-675 residue or deletion of the molecule’s C-terminus prevents Notch activation and reduces cell motility and tumor growth [28].

Previous studies demonstrated high levels of ASPH expression in clinically aggressive malignancies, such as those originating from the gastrointestinal tract, lung, liver, pancreas, breast, or central nervous system [29,33,34,35,36,37]. Aberrantly increased Notch activity has been implicated in many malignancies, including chondrosarcoma [19]. Furthermore, high levels of ASPH expression were shown to correlate with worse clinical outcomes [29,38], including abbreviated survival [36,39]. In contrast, most normal cells and tissues express very low or non-detectable levels of ASPH [34]. Similarly, preliminary studies also suggest that high-grade conventional chondrosarcomas express higher levels of ASPH than low-grade chondrosarcomas [40]. In light of the well-documented roles of Notch in many malignancies, including chondrosarcoma, ASPH could potentially serve as an excellent target for chondrosarcoma diagnostics and therapeutics [41,42,43,44].

One evolving approach for cancer therapeutics is the use of small molecule inhibitors that target critical signaling pathways utilized in the growth and survival of malignant cells, including inhibitors of tyrosine and serine/threonine kinases, proteosomes, apoptosis, or matrix metalloproteinases [45]. Of note, a small molecule inhibitor of ADAM (INCB7839), designed to release the extracellular domain of Notch, was shown to be an effective anti-cancer agent in epithelial neoplasms [46,47]. Regarding ASPH, previous studies led to the design and characterization of unique small molecule inhibitors that fit in the pocket of ASPH’s catalytic domain and diminish or abolish its enzymatic (hydroxylase) activity [27,35,36,38,48,49]. The ASPH small molecule inhibitor 1182 (SMI1182) was shown to inhibit ASPH’s functions linked to Notch activation [36,48,49] and produce anti-tumor effects in experimental models of cholangiocarcinoma [48], hepatocellular carcinoma [38], pancreatic carcinoma [35], breast cancer [27], glioblastoma [36], and more recently, chondrosarcoma [40]. However, despite encouraging results, none of the studies demonstrated complete cancer cell killing with SMI1182 used as a monotherapy. The preliminary finding that the combined use of SMI1182 with DOX in an experimental breast cancer model was more effective than SMI1182 monotherapy [50] inspired further investigation of this concept in relation to chondrosarcoma.

We hypothesized that SMI1182 could potentially be used as an adjuvant chemotherapeutic agent to enhance DOX-mediated CS killing. In addition, we postulated that the simultaneous targeting of Notch and mTOR would further enhance the killing of CS cells. These concepts have clinical relevance because if SMI1182 and BEZ235, a potent inhibitor of PI3K/mTOR, could additively increase the chemotherapeutic effects of DOX, then more effective CS treatment could be provided at lower, less toxic doses of DOX. Herein, we report results from in vitro experimental models to assess the potential for future in vivo therapeutics to improve clinical outcomes in patients with CS.

## 2. Materials and Methods

The studies utilized three mycoplasma-free human CS cell lines: CS1 (RRID: CVCL_T022), CDS11 (RRID: CVCL_WJ30), and CDS17 (T-CDS17: CVCL_WJ32; CDS17: CVCL_WJ31). The CS1 cell line originated from a high-grade (Grade 3) conventional CS tumor in an untreated patient with metastatic disease [51]. CDS11 cells correspond to slow-growing, less invasive secondary conventional CS [12,52]. CDS17 cells are de-differentiated chondrosarcoma cells with a more invasive phenotype compared with CDS11 [52]. All three cell lines were authenticated using short tandem repeat profiling, and all studies were performed with cells passaged 10 or fewer times. CS1 cells were maintained in Roswell Park Memorial Institute (RPMI) 1640 medium (Cytiva/Hyclone, Logan, UT, USA). CDS11 and CDS17 cells were grown in Dulbecco’s Modified Eagle’s Medium (DMEM) with high glucose (Cytiva/Hyclone, Logan, UT, USA). Culture medium was supplemented with 5% fetal bovine serum (FBS) and 2 mM L-Glutamine (Corning, Glendale, AZ, USA), and all in vitro experiments were conducted at 37 °C in a standard humidified 5% CO_2_ incubator.

**Figure 2:**
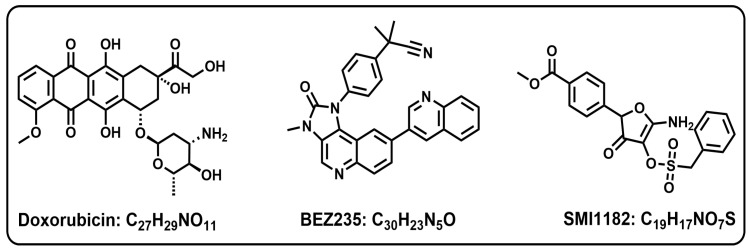
Chemical structures and formulas of Doxorubicin, BEZ235, and SMI1182.

The investigations were focused on CS1 and CDS11 cells to compare responses between intermediate and high-grade conventional chondrosarcomas. To study the effects of DOX, SMI1182, BEZ235 (see chemical structures in Figure 2), and combination treatments, 24 h old sub-confluent CS cultures were treated with vehicle (DMSO; negative control), 0.0001–10 µM DOX (Pfizer Inc., New York, NY, USA), 0–100 μM SMI1182, or 0.002–2.0 μM BEZ235 by direct addition to the medium. We compared the dose-related effects of monotherapy with the effects of dose-range monotherapy plus a fixed-dose second or third compound (combination therapy). After 48 h of treatment, the cultures were analyzed for cell viability, cytotoxicity, morphology, ASPH immunoreactivity, directional motility, mRNA transcripts related to Notch and insulin, insulin-like growth factor (IGF)/insulin receptor substrate (IRS) networks, and Akt-mTOR pathway signaling. The rationale for these approaches was that previous studies linked ASPH regulation and function to the activation of insulin/IGF-Akt and Notch in various cancers [29,53,54,55].

### 2.1. Cell Viability and Cytotoxicity Assays

Cell viability and metabolic function were measured with the 3-(4,5-dimethylthiazol-2-yl)-2,5-diphenyltetrazolium bromide (MTT) (#M5655, Sigma-Aldrich, St. Louis, MO, USA) assay [56,57,58]. Cell density was assessed by Hoechst 33342 (H3570, Invitrogen, Carlsbad, CA, USA) fluorescence. Cytotoxicity was measured with the CyQUANT Cytotoxicity Assay Kit (G6PD Release) (Thermo Fisher Scientific, Bedford, MA, USA), which is based on glucose-6-phosphate dehydrogenase release into the culture supernatant. All three assays were performed in 96-well cultures with 3000 viable (Trypan blue-excluded) CS cells seeded in 100 µL of culture medium. The MTT assay was performed by adding 10 µL/well of freshly prepared MTT solution (5 mg/mL in MEM without phenol red) and incubating the plate for 20 min at 37 °C in a 5% CO_2_ atmosphere. After aspirating the culture medium, the substrate was eluted by adding 100 µL of acidic isopropanol elution buffer (0.04 mol/L HCl/isopropanol) and 5 min of gentle platform agitation at room temperature. Absorbances were measured at 540 nm in a Spectra-Max M5 Multimode Plate Reader (Molecular Devices, Sunnyvale, CA, USA) [56,57,58]. To measure cell number in the same wells as MTT activity, the eluate buffer was replaced with 50 µL of 10 µg/mL Hoechst 33342 dye in phosphate-buffered saline (PBS). Fluorescence intensity (Ex360nm/Em460nm) was measured in a Spectra-Max M5 after 5 min of room temperature incubation with light-shielding. Cytotoxicity was quantified in 50 µL of culture supernatant added to CyQUANT assay solution and incubated for 30 min. Fluorescence intensity (Ex530nm/Em590nm) was measured in a Spectra-Max M5.

### 2.2. Sample Preparation for Immunoassays

CS cells seeded into 6-well plates (1 × 10^5^/mL) were treated for 48 h with vehicle (DMSO), DOX, SMI1182, BEZ235, or combinations. For protein assays, the cells were harvested in weak lysis buffer (50 mM Tris (pH 7.5), 150 mM NaCl, 5 mM EDTA (pH 8.0), 50 mM NaF, 0.1% Triton X-100) containing protease inhibitor cocktail (1mM PMSF, 0.1 mM TPCK, 2 µg/mL aprotinin, 2 µg/mL pepstatin A, 1 µg/mL leupeptin). After centrifuging the homogenates at 14,000× *g* rpm for 10 min at 4 °C, the supernatant fractions were divided and stored at −80 °C. Prior to freezing, small aliquots were set aside to measure protein concentration with the bicinchoninic acid (BCA) assay. Proteins harvested from 6-well cultures and treated as described above were used for Western blot analysis and enzyme-linked immunosorbent assays (ELISAs) to measure immunoreactivity to ASPH (two antibodies: A85G6 and FB50), vimentin, GAPDH, β-Actin, and large acidic ribonuclear protein (RPLPO), as previously described [28,59,60,61]. (See Table 1 for antibody sources, characteristics, validations, and conditions of use. RPLPO served as a loading control due to its linear correlation with protein content [62].)

### 2.3. Western Blot Analysis

Western blot analysis was performed by fractionating protein homogenates (30 μg samples) in 10% SDS-PAGE gels along with pre-stained molecular weight standards (Precision Plus Protein Dual Color Standards, Bio-Rad Laboratories, Hercules, CA, USA; Catalog #161-0374). The samples were electroblot transferred to polyvinylidene difluoride (PVDF) membranes (Bio-Rad Laboratories, Hercules, CA, USA) and incubated with SuperBlock-TBS (Thermo Fisher, Bedford, MA, USA) to mask non-specific binding sites. The membranes were then incubated with primary antibodies overnight at 4 °C with gentle platform agitation. Immunoreactivity was detected with horseradish peroxidase (HRP)-conjugated secondary antibody and Femto-chemiluminescence reagent (Thermo Fisher Scientific; Prod #34096). Immunoreactivity was imaged using the Intelligent Dark Box II (FUJIFILM, Tokyo, Japan). The original, uncropped Western blot membrane can be found in Supplementary Figure S1.

### 2.4. Enzyme-Linked Immunosorbent Assay (ELISA)

Triplicate 50 ng aliquots of protein were adsorbed to the bottom surfaces of enzyme immunoassay (EIA) MaxiSorp flat-bottom 96-well plates (Thermo Fisher Scientific; Catalog #436110) by overnight incubation at 4 °C. Non-specific sites were masked with SuperBlock-TBS (Thermo Fisher Scientific; Catalog #37535). Primary antibodies were incubated overnight at 4 °C. After thorough rinsing of the wells, the samples were sequentially incubated with horseradish peroxidase (HRP)-conjugated secondary antibody and Amplex UltraRed fluorophore with TBS rinses between steps. Fluorescence intensity (Ex530nm/Em590nm) was measured in a Spectra-Max M5 instrument. Negative controls included the omission of the primary antibody, secondary antibody, or Amplex UltraRed. The results were normalized to RPLPO as a sample loading control because previous studies showed that the levels of RPLPO immunoreactivity correlate with protein content and do not vary with experimental conditions [61]. In contrast, GAPDH, which is regulated by insulin/IGF stimulation [63] and oxidative stress [64], was found to vary with cytotoxic agent treatment and, therefore, could not be used as a loading control.

### 2.5. Immunocytochemistry

Cytospin preparations of cultured cells were generated using a Shandon Cytospin 3 Cytology Centrifuge with Rotor (Marshall Scientific, Hampton, NH, USA). Cells grown in 6-well plates were trypsinized and re-suspended to 0.5 × 10^6^ cells/mL in culture medium containing 0.5% FBS to prevent non-specific lysis. Approximately 0.5 × 10^5^ cells were centrifuged onto Plus-charged glass microscope slides at 600× *g* rpm for 5 min at RT, and then immediately fixed in 10% neutral buffered formalin. Replicate slides were stained with crystal violet (Thermo Scientific Chemicals, Haverhill, MA, USA) to examine cell morphology or immunostained with the FB50-ASPH monoclonal antibody (Laboratory-made). Immunoreactivity was detected with the ImmPRESS peroxidase polymer detection reagents (Vector Laboratories, Newark, CA, USA) and diaminobenzidine (DAB) as the chromogen according to the manufacturer’s protocol. The immunostained cells were lightly counterstained with Gill’s Hematoxylin 2, dehydrated through graded ethanol solutions, and preserved under coverglass with Cytoseal 60 mounting media (Epredia, Kalamazoo, MI, USA; REF# 8310-4).

### 2.6. Motility Assay

Directional motility was measured using the ATP Luminescence-Based Motility/Invasion (ALMI) assay [65]. This assay was constructed with blind well chambers (Neuro Probe, Gaithersburg, MD, USA) divided into upper and lower segments using 13 mm diameter, 8 μm pore diameter polycarbonate membranes. Motility was measured using uncoated membranes. The motility/invasion assay was assembled by placing culture medium (200 µL) containing 1% FBS as the trophic factor in the blind well (lower part of the chamber below the membrane) and 1.0 × 10⁵ viable cells in 100 µL of 0.1% FBS medium in the upper chamber. The assays were incubated for 30 min at 37 °C in a standard CO_2_ cell culture incubator. Results were analyzed by measuring ATP luminescence in the cells remaining in the upper chamber (non-migrated), distributed on the undersurface of the membrane (migrated adherent), and distributed in the lower chamber (migrated non-adherent). ATP content with measured with ATP-Lite reagents (Perkin-Elmer, Waltham, MA, USA), as previously described [65]. Luminescence was quantified in a TopCount NXT Microplate Scintillation and Luminescence Counter (GMI, Ramsey, MN USA). The percentages of non-motile, motile adherent, and motile non-adherent cells in replicate assays were calculated and used for statistical analysis.

### 2.7. Quantigene 2.0 RNA Multiplex Assay

Total RNA was extracted from 6-well CS cultures using QIAzol Lysis Reagent (Germantown, MD USA). Messenger RNA transcripts corresponding to the human insulin/Notch pathway signaling molecules were quantified with a custom Quantigene 2.0 Multiplex (QGP) Assay (Affymetrix Inc., Santa Clara, CA, USA). RPL13a served as the internal control gene for normalizing results. Cooperative hybridization and quantification were performed following the manufacturer’s protocol. Briefly, the assay allows for direct mRNA quantification using xMAP Luminex beads and reporter signal amplification. A working bead mix containing lysis mixture, blocking reagent, capture beads, and a 2.0 probe set was prepared and distributed in a 96-well format plate. Total RNA (1 µg) was added to the sample wells and incubated overnight with the xMAP fluorescent beads. Sterile nuclease-free water was used as the negative control. The samples were first incubated with a set of oligonucleotide probes (pre-amplifier, amplifier, and biotin-label), followed by streptavidin-conjugated R-Phycoerythrin (SAPE). The resulting fluorescent signals were detected with a Luminex MAGPIX instrument (Diasorin, Austin, TX, USA). MAGPIX calibration and verification standards were used throughout, ensuring the levels of SAPE fluorescence were proportional to RNA transcript abundance captured by the beads. After subtracting the probe-related background from the target median fluorescence intensity (MFI), the results were normalized to RPL13a.

### 2.8. Multiplex ELISAs (Akt-mTOR Pathway)

We used 11-Plex MILLIPLEX Akt/mTOR Total and Phosphoprotein Magnetic Bead Kits (MilliporeSigma, Burlington, MA, USA) to evaluate the effects of treatment on mTOR signaling mechanisms in CS cells (Table 2). Multiplex ELISAs were performed according to the manufacturer’s protocol except that the reactions were optimized by using 12.5 µg protein/sample. Following incubation with antibody-bound beads, immunoreactivity in the captured antigens was detected with biotinylated secondary antibodies and phycoerythrin-conjugated streptavidin and measured in a MAGPIX with xPONENT software (https://int.diasorin.com/en/luminex-ltg/reagents-accessories/software). Standard curves were generated for each analyte.

### 2.9. Data Analysis

All assays were repeated at least 3 times, with 3 or 4 technical and experimental replicates per sample. Violin plots depict the distribution of results with the median (horizontal bars) and 95% confidence interval limits. The data were analyzed by *t*-test or Analysis of Variance (ANOVA) with corrections for multiple comparisons. Statistical analyses and graphical presentation of results were performed using GraphPad Prism 10.4 (GraphPad Software Inc., San Diego, CA, USA).

### 2.10. Reagent Sources

The ELISA MaxiSorp 96-well plates, bicinchoninic acid (BCA) reagents, horseradish peroxidase (HRP)-conjugated secondary antibodies, and Superblock (TBS) were purchased from Thermo-Fisher Scientific (Bedford, MA, USA). The soluble fluorophores, Amplex UltraRed and 4-Methylumbelliferyl phosphate (4-MUP), were from Life Technologies (Carlsbad, CA, USA). All other fine reagents were purchased from CalBiochem/Millipore Sigma (Burlington, MA, USA), Pierce Chemical (Dallas, TX, USA), or Sigma-Aldrich Co. (St. Louis, MO, USA). SMI1182 was synthesized, crystallized, and validated using NMR, mass spectrometry, and thin-layer chromatography [66] (see Figure S1).

## 3. Results

### 3.1. ASPH Immunoreactivity Detected in CS Cells

**Figure 3:**
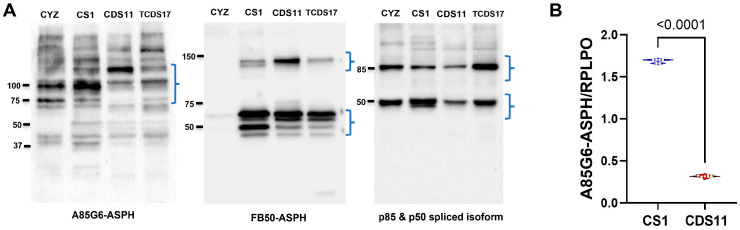
(**A**) The A85G6 or FB50 monoclonal antibody, which binds to the C-terminal or N-terminal region of ASPH, was used for Western blot analysis of CYZ (neuroblastoma; positive control), CS1, CDS11, and TCDS17 cells. The same blot was stripped and re-probed. The third probing with polyclonal antibodies to p85 served as a loading control. The positions of molecular weight standards are indicated. Brackets mark the expected positions of A85G6-ASPH, FB50-ASPH/Humbug, and p85 together with the p50 subunit. (**B**) ELISA results comparing levels of A85G6-ASPH immunoreactivity in CS1 and CDS11 cells. The levels of immunoreactivity were normalized to RPLPO measured in the same samples. Intergroup comparisons were made by *t*-test.

Western blot analysis was performed with two ASPH monoclonal antibodies, FB50 and A85G6. FB50 binds to the N-terminal region of ASPH, which overlaps with the sequence for Humbug [67,68], a truncated form of ASPH lacking the C-terminal catalytic domain [69,70,71]. A85G6 binds to the C-terminus of ASPH [68], which contains a unique catalytic domain for Notch and Jagged hydroxylation [67,69]. Western blot analysis detected ASPH immunoreactivity in CS1 (Grade 3 conventional chondrosarcoma), CDS11 (Grade 2 conventional chondrosarcoma), CDS17 (de-differentiated chondrosarcoma), and CYZ/PNET2 neuroblastoma cells (positive control) (Figure 3A). A85G6 detected the expected ~86kD full-length ASPH protein, as well as super-shifted, likely phosphorylated forms, and cleavage products in all four cell lines (Figure 3A, left panel). FB50 detected abundant ASPH immunoreactivity in the three CS cell lines but very low levels in CYZ/PNET2 cells (Figure 3A, middle panel). The dominant FB50-positive band corresponds to Humbug. Re-probing the blot with antibodies to the p85-subunit of PI3 Kinase detected p85 and its p50 spliced variant and demonstrated comparable sample protein loading in each lane [72] (Figure 3A, right panel). ELISA studies confirmed the presence of A85G6 ASPH immunoreactivity in CS1 and CDS11 cells and more clearly demonstrated higher levels of A85G6-ASPH immunoreactivity in CS1 compared with CDS11 cells (Figure 3B).

### 3.2. Mechanistic and Functional Differences Between CS1 and CDS11 Cells

**Figure 4:**
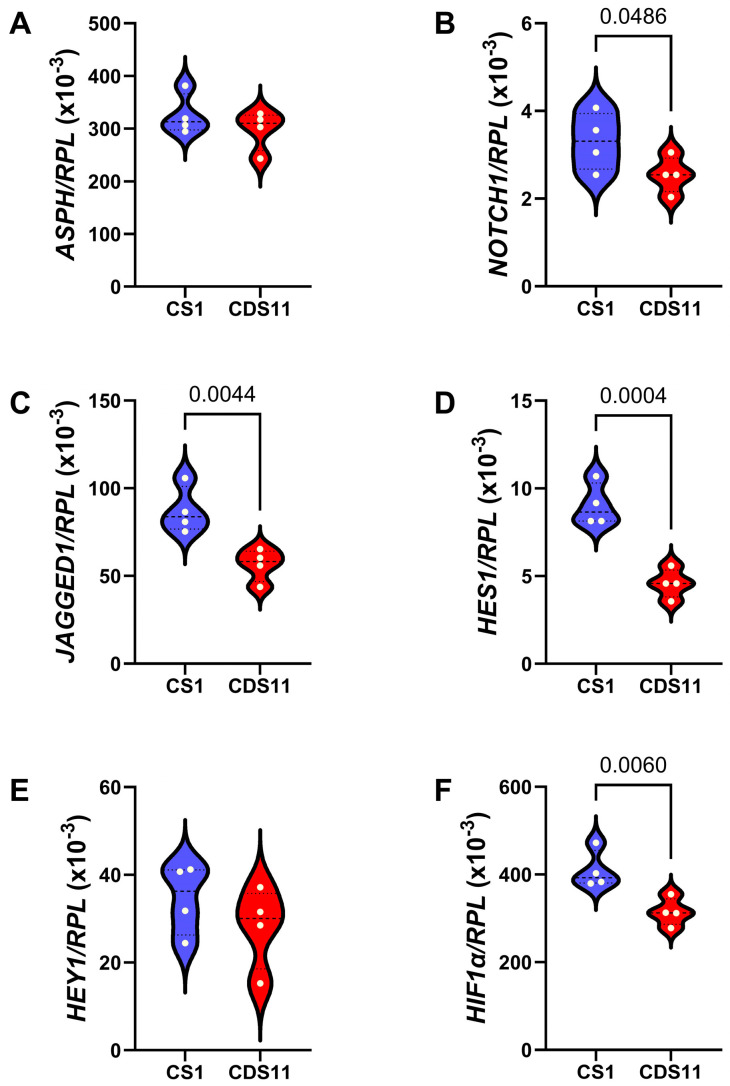
ASPH and Notch network gene expression in Grade 3 versus Grade 2 conventional chondrosarcoma. CS1 (Grade 3) and CDS11 (Grade 2) cells were analyzed for (**A**) *ASPH*, (**B**) *NOTCH1*, (**C**) *JAGGED1*, (**D**) *HES1*, (**E**) *HEY1,* and (**F**) *HIF1α* mRNA expression using a multiplex bead-based RNA hybridization protocol with results normalized to Ribosomal Protein L13a (RPL). Inter-group comparisons were made by *t*-test analysis. Significant *p*-values (*p* ≤ 0.05) are shown in the panels.

Since higher levels of ASPH have been associated with higher-grade tumors [31,35,36,42,73,74,75,76] and abbreviated survival and post-treatment, disease-free intervals [31,36], we compared the CS1 and CDS11 cells with respect to directional motility (Figure S2), mRNA expression of insulin/IGF/IRS pathway genes that regulate ASPH (Figure S3), and mRNA expression of Notch pathway molecules (Figure 4). Using the ATP-Lite Motility assay, we detected similar percentages of motile adherent, motile non-adherent, non-motile, and total motile populations in CS1 versus CDS11 cultures (Figure S2). Multiplex RNA hybridization studies demonstrated that *INSULIN, IGF1*, and *IGF2* were expressed at low levels in CS cells, *IGF2R* was more abundantly expressed than *INSULIN-R* and *IGF1R*, and *IRS1* was 10-fold more abundant than *IRS2* and *IRS4* (Figure S3). Inter-group statistical comparisons revealed that CDS11 cells had significantly (*p* ≤ 0.05) or statistical trendwise (0.10 < *p* < 0.05) lower expression of *INSULIN-R* (Figure S2D), *IGF2R* (Figure S2F), and IRS1 (Figure S2G) but higher levels of *INSULIN* (Figure S1A) compared with CS1 cells. The other five *INSULIN/IGF/IRS* pathway genes were similarly expressed in CS1 and CDS11 cells (Figures S2B,C,E,H,I). Concerning Notch pathway mRNA transcripts, CDS11 cells expressed significantly lower levels of *NOTCH1* (Figure 4B), *JAGGED1* (Figure 4C), *HES1* (Figure 4D), and *HIF1a* (Figure 4F) compared to CS1 cells. In contrast, there were no significant inter-group differences in mRNA expression of *ASPH* (Figure 4A) or *HEY1* (Figure 4E).

### 3.3. DOX, BEZ235, and SMI1182 Monotherapy and Combination Therapy Effects on Metabolic Activity, Cell Viability, and Cytotoxicity

This segment of the study examined CS1 and CDS11 dose-responses to DOX, BEZ, and SMI1182 monotherapy and combination therapy using the MTT activity, H33342 fluorescence (index cell number), and CYQUANT assays. All assays were performed in 96-well cultures that were treated for 48 h with dose ranges of DOX (0.0001–1 µM), BEZ235 (BEZ) (0.002–2.0 μM), or SMI1182 (0 to 100 µM), or with vehicle (DMSO control). The effects of combination therapy were assessed by combining the dose range of one compound with a fixed dose of one or two of the other compounds. In addition to dose–response curves, treatment effects were compared using area-under-curve (AUC) analysis and one-way ANOVA tests. The MTT assay provides a measure of mitochondrial function, proliferation, and cell viability. However, under conditions of mild oxidative stress, MTT activity can increase, potentially leading to erroneous interpretations about cellular responses, e.g., proliferation [58]. To complement the MTT studies, the cells were also stained with H33342 dye, which labels DNA; H33342 fluorescence is linearly correlated with cell number. Dead or dying cells detach from the plate and thus are not counted/measured in the assay. Cellular cytotoxic responses were evaluated by measuring G6PD release using the CYQUANT assay.

**Figure 5:**
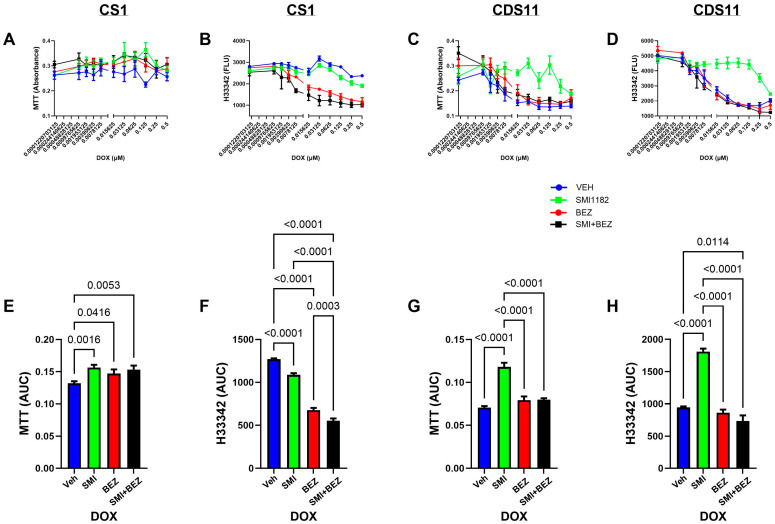
Dose effects of DOX+Vehicle, DOX+SMI1182, DOX+BEZ, or DOX+SMI+BEZ on MTT activity (**A**,**C**) and H33342 fluorescence (viability; **B**,**D**) in CS1 (**A**,**B**) and CDS11 (**C**,**D**) cells. The assays were performed with cells seeded in 96-well micro-cultures and treated for 48 h with dose ranges of DOX plus fixed doses of SMI1182 (0.5 µM), BEZ (2 µM), or SMI+BEZ. Panels (**E**–**H**) depict area-under-curve analysis results corresponding to the curves in Panels (**A**–**D**), respectively. Data points and bar graphs show mean ± S.D of results of 4 replicate cultures. Inter-group differences were compared by ANOVA with post hoc tests. Significant *p*-values (*p* ≤ 0.05) are shown in the panels.

DOX (Figure 5): In CS1 cells, DOX monotherapy had minimal effects on MTT activity or H33342 fluorescence over most of the dose range tested (Figure 5A,B). Co-treatment with SMI1182, BEZ, or SMI1182+BEZ slightly increased MTT activity but profoundly reduced H33342 relative to vehicle, as demonstrated by ANOVA tests of the AUC analyses (Figure 5E,F) (MTT: F = 4.465; *p* = 0.008; H33342: F = 231.6; *p* < 0.0001) with post hoc Tukey tests. The H33342 results showed that cell loss was greatest in cultures co-treated with DOX+SMI+BEZ, followed by DOX+BEZ. In contrast, CDS11 cells exhibited similar dose-dependent reductions in MTT and H33342 following treatment with DOX monotherapy, DOX+BEZ, or DOX+BEZ+SMI1182 (Figure 5C,D,G,H). However, co-treatment with DOX plus SMI1182 was protective in maintaining relatively high levels of MTT and H33342, except at the highest doses of DOX. ANOVA tests of the AUC analyses demonstrated significant inter-group differences for both MTT (F = 37.35, *p* < 0.0001) and H33342 (F = 37.35, *p* < 0.0001).

**Figure 6:**
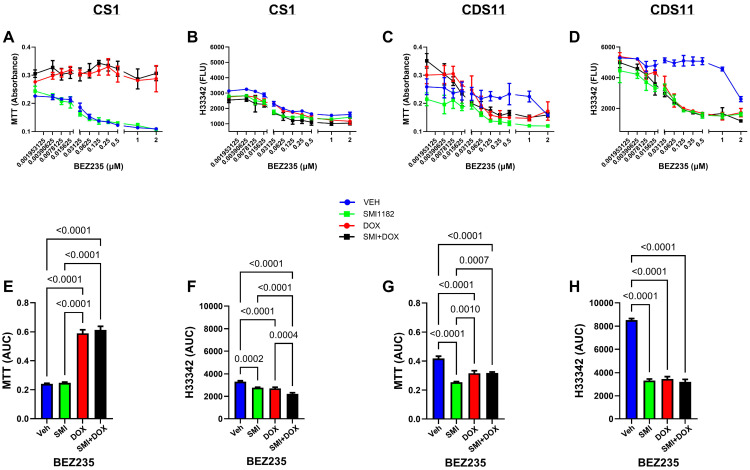
Dose effects of BEZ+Vehicle (Veh), BEZ+SMI1182, BEZ+DOX, or BEZ+SMI+DOX on MTT activity (**A**,**C**) and H33342 fluorescence (viability; **B**,**D**) in CS1 (**A**,**B**) and CDS11 (**C**,**D**) cells. The assays were performed with cells seeded in 96-well micro-cultures and treated for 48 h with dose ranges of BEZ plus fixed doses of SMI1182 (0.5 µM), DOX (0.05 µM), or SMI+DOX. Panels (**E**–**H**) depict area-under-curve analysis results corresponding to the curves in Panels (**A**–**D**), respectively. Data points and bar graphs show the mean ± S.D. of results from 4 replicate cultures. Inter-group differences were compared by ANOVA with post hoc Tukey tests. Significant *p*-values (*p* ≤ 0.05) are shown in the panels.

BEZ (Figure 6): CS1 cells treated with BEZ or BEZ+SMI1182 exhibited similar dose-dependent reductions in MTT activity, whereas co-treatment with BEZ+DOX or BEZ+DOX+SMI resulted in sustained high levels of MTT over the full BEZ dose range, as confirmed by AUC analysis (Figure 6A,E). Correspondingly, ANOVA tests were significant for the inter-group differences in MTT (F = 132.7, *p* < 0.0001) and H33342 (F = 26.12, *p* < 00001). In contrast, in CS1 cells treated with BEZ alone or in combination with SMI1182, DOX, or DOX+SMI1182, H33342 fluorescence (cell viability) progressively declined (Figure 6B), with significant additive effects of the co-treatments, as demonstrated by AUC analysis (Figure 6F). CDS11 cells treated with BEZ alone exhibited reduced MTT activity at the lowest doses but minimal further reduction except at the highest doses (1–2 µM) (Figure 6C). Co-treatment with SMI1182 reduced MTT such that the mean AUC was significantly below the other three treatments (F = 30.17, *p* < 0.0001) (Figure 6C,G). BEZ+DOX or BEZ+DOX+SMI1182 caused similar dose-dependent and overall reductions in MTT relative to BEZ-only treatment (Figure 6C,G). Regarding H33342, BEZ-only had a minimal effect on CDS11 cell viability, but the addition of SMI1182, DOX, or DOX+SMI1182 sharply reduced cell viability by 50% or more (F = 221.6, *p* < 0.0001) (Figure 6D,H).

**Figure 7:**
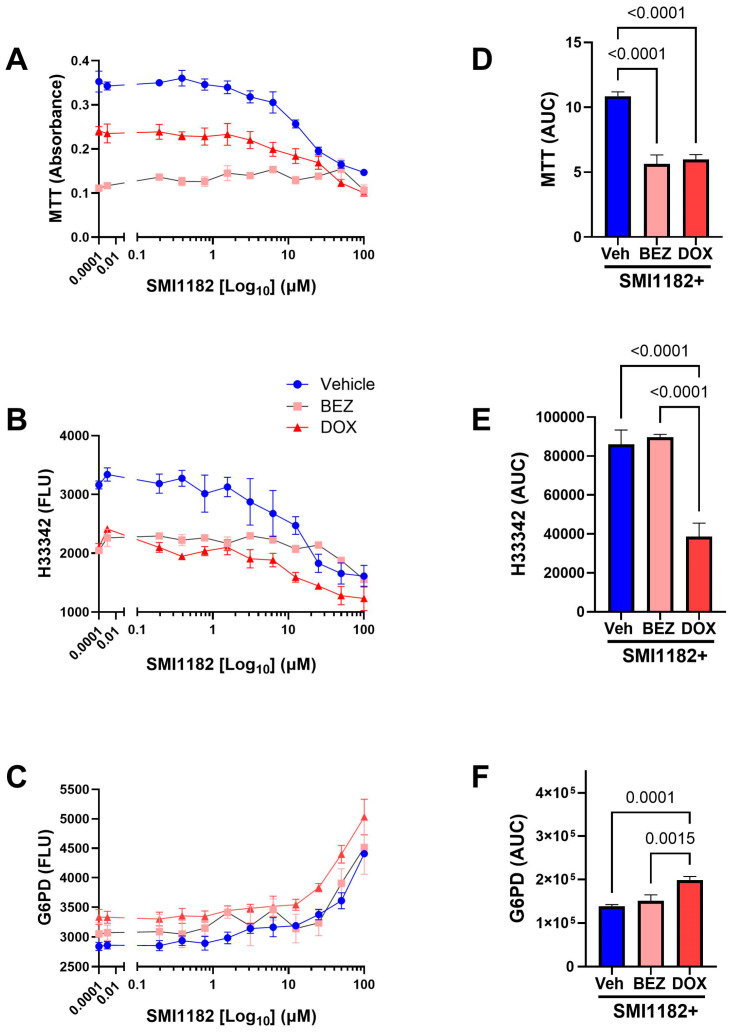
Dose effects of SMI1182 plus vehicle, BEZ, or DOX on MTT activity, H33342 fluorescence (viability), and G6PD release (cytotoxicity). CS1 96-well micro-cultures were treated with a dose range of SMI1182 and co-treated with vehicle or fixed doses of BEZ or DOX for 48 h. (**A**) MTT, (**B**) H33342, and (**C**) G6PD assay results were measured in 4 replicate cultures per data point. Panels (**D**–**F**) depict area-under-curve analyses. Data points and bar graphs show mean ± S.D. of results. by ANOVA with post hoc Tukey tests. Significant *p*-values (*p* ≤ 0.05) are shown in the panels.

SMI1182 (Figure 7): Previous studies showed that SMI1182 monotherapy functions by reducing invasive growth and cell motility [77,78]. In the present study, the treatment of CS1 cells with 10 µM or lower concentrations of SMI1182 caused only modest reductions in MTT activity (Figure 7A) and H33342 fluorescence (Figure 7B) and minimal increases in G6PD release (cytotoxicity) (Figure 7C). However, SMI1182 treatments above 10 µM sharply reduced viability and increased cytotoxicity. Dual SMI1182+BEZ or SMI1182+DOX significantly reduced MTT activity relative to SMI1182 monotherapy, as shown in both the dose–response curves and AUC analyses (F = 34.62, *p* < 0.0001) (Figure 7A,D). In addition, SMI1182 + DOX reduced H33342 (F = 23.30, *p* < 0.0001) (Figure 7B,E) and increased cytotoxicity (F = 10.69, *p* = 0.0003) (Figure 7C,F) compared with SMI1182 monotherapy and SMI1182+BEZ. Although at lower doses BEZ co-treatment reduced CS1 cell viability, at higher doses there was no significant difference relative to SMI1182 alone, accounting for the similar AUC calculated results. Correspondingly, there were no significant effects of SMI1182+BEZ on G6PD release relative to SMI1182 treatment alone (Figure 7C,F).

### 3.4. Effects of SMI1182, DOX, and BEZ on CS Morphology

**Figure 8:**
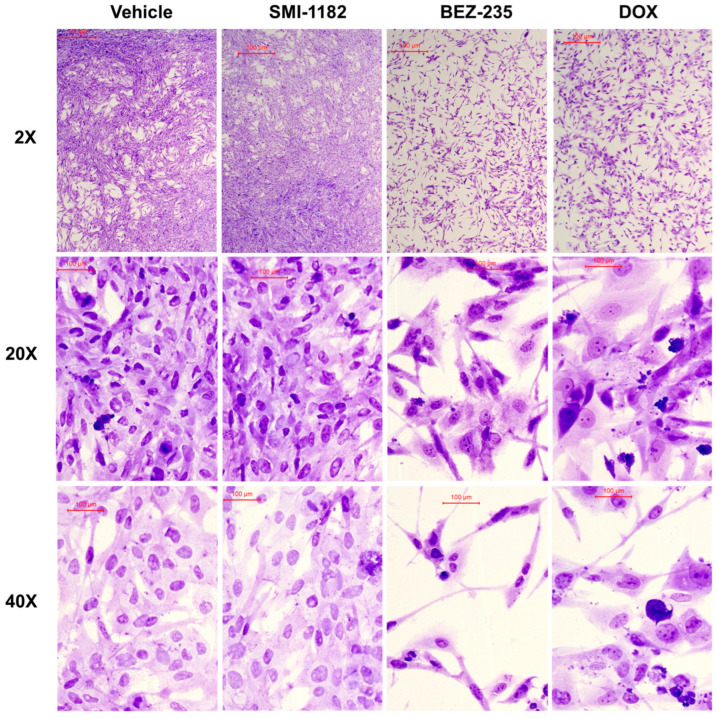
Cytomorphological effects of vehicle, SMI1182, BEZ235, or DOX treatment of CS1 chamber slide cultures. CS1 cells were treated for 48 h, then fixed in formalin, stained with crystal violet, and examined by light microscopy. Representative areas of the cultures were photographed with the 2×, 20×, or 40× objective. Scale bars (100 µm) are included in each panel. Note the similarly high cell densities and relatively uniform cellular and nuclear morphologies in cultures treated with vehicle or SMI1182, versus lowered cell densities and greater variation in nuclear and cellular sizes and the tapered, spindled appearance of cells in BEZ235- and DOX-treated cultures.

**Figure 9:**
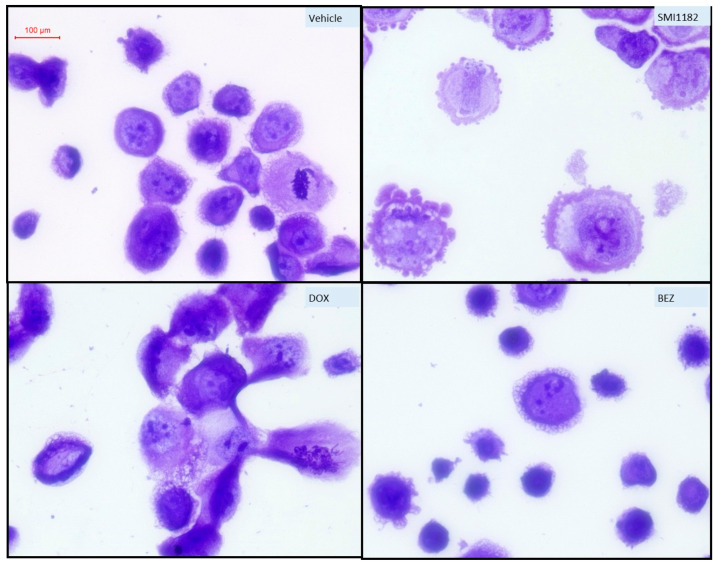
Cytomorphological effects of vehicle, SMI1182, DOX, or BEZ235 treatment of CS1 cells. After 48 h of treatment in 6-well plates, the cells were harvested and used to generate cytospin preparations, which were fixed in formalin, stained with crystal violet, and examined by light microscopy. (**Upper Left**) Vehicle/control-treated cells exhibited variation in cell size, prominent nucleoli, and nuclear condensation. (**Upper Right**) SMI1182 treatment resulted in prominent cytoplasmic surface blebbing, cellular enlargement, and both nuclear and cytoplasmic vacuolation. (**Lower Left**) DOX treatment increased nucleolar prominence and cytoplasmic vacuolation. (**Lower Right**) BEZ caused abundant nuclear condensation and necrosis, as well as conspicuous fine, hair-like surface processes.

Crystal violet-stained chamber slide cultures (Figure 8) and cytospin preparations (Figure 9) were used to evaluate cellular morphological responses to 48 h of treatment with vehicle, SMI1182, BEZ, or DOX. The chamber slides showed that the vehicle- and SMI1182-treated cultures grew to confluence with high cellular densities, and they exhibited similar nuclear and cytoplasmic morphologies (Figure 8). In contrast, parallel cultures treated with BEZ or DOX exhibited reduced cell densities, increased nuclear hyperchromasia (dark, densely stained), numerous cells with narrow, tapered morphologies, and scattered cellular debris (Figure 8). In addition, DOX increased the variability in cell size manifested by irregular nuclear and cytoplasmic enlargements, whereas BEZ-treated cells often exhibited small shrunken nuclei (pre-apoptotic).

Cytospin preparations were generated by trypsin-dissociating the cultures and centrifuging the cells onto glass slides in a 5 mm focused area. This approach allows the visualization of individual cells apart from the monolayers. Control (vehicle-treated) cells had pleomorphic polygonal morphologies with high nuclear-to-cytoplasmic ratios, variability in cell size, scattered cells with nuclear condensation (pyknosis), and readily detected nuclear mitoses (Figure 9, Upper Left). SMI1182 treatment resulted in conspicuous cellular enlargement and surface blebbing, nucleolar prominence, and cytoplasmic vacuolation (Figure 9, Upper Right). DOX increased cell size, shape variability, nuclear condensation, apoptosis, cellular fragmentation, and atypical nuclear mitoses (Figure 9, Lower Left). BEZ treatment resulted in pronounced cellular/nuclear condensation (pre-apoptotic stage), modest surface blebbing, and cells with abundant fine hair-like surface processes (Figure 9, Lower Right). Combined SMI1182+DOX, SMI1182+BEZ, or BEZ+DOX treatments produced overlapping cytomorphological changes corresponding to the effects of each drug.

### 3.5. Effects of SMI1182, DOX, and BEZ on ASPH Immunoreactivity

**Figure 10:**
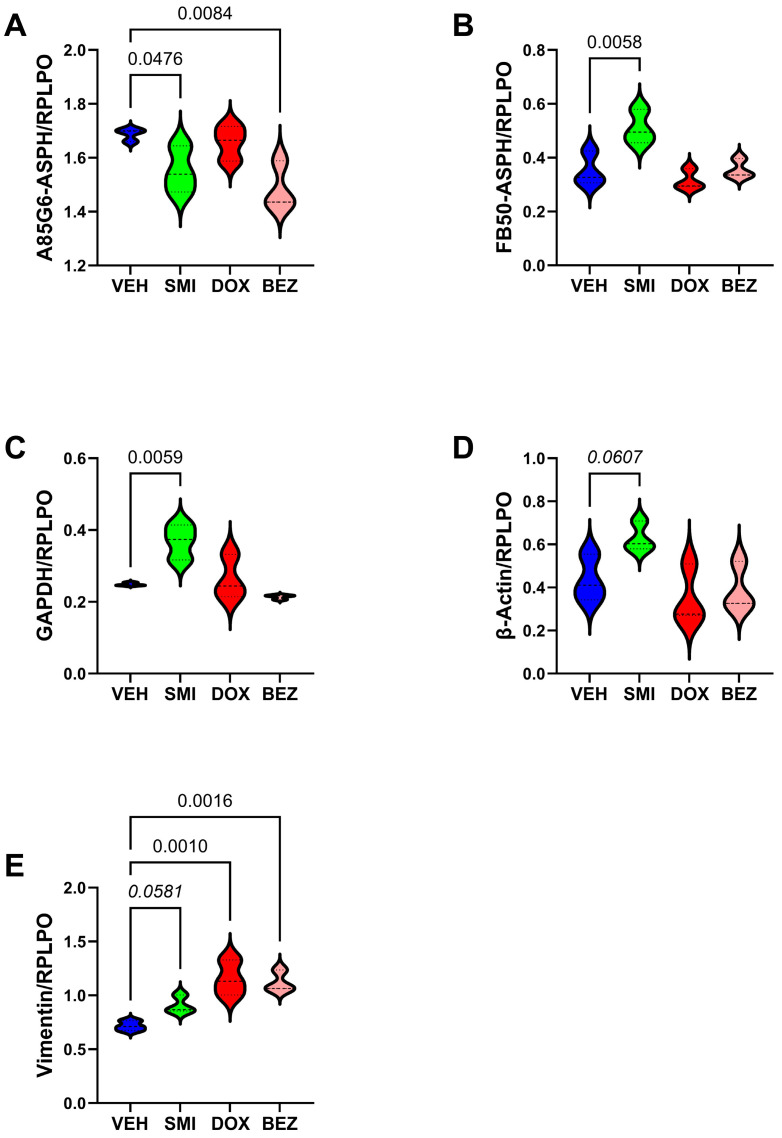
ELISA results following treatment of CS1 cells with SMI1182, DOX, or BEZ. Immunoreactivity to (**A**) A85G6-ASPH, (**B**) FB50-ASPH, (**C**) GAPDH, (**D**) β-Actin, and (**E**) vimentin was measured in 4 replicate 50 ng protein samples by ELISA, with results normalized to RPLPO. Violin plots depicting the median (mid-level line) and 95% confidence interval limits (upper and lower lines). Inter-group statistical comparisons were made using one-way ANOVA with post hoc Tukey tests. Significant *p*-values (*p* ≤ 0.05) are shown in the panels. Statistical trend effects (0.10 < *p* < 0.05) are indicated with italic font.

**Figure 11:**
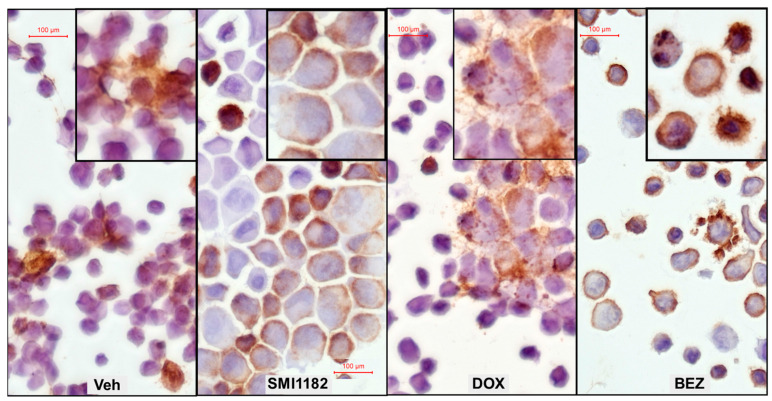
Altered cellular distributions of ASPH immunoreactivity in CS1 cells treated with vehicle (Veh), 3.125 µM SMI1182 (SMI1182), 0.05 µM DOX, or 2 µM BEZ for 48 h. Cytospin preparations were immunostained with FB50-ASPH monoclonal antibody, and immunoreactivity was revealed with biotinylated secondary antibody, horseradish peroxidase-conjugated streptavidin, and DAB (brown precipitate). All images were obtained at 400× magnification. Insets show higher magnification images of FB50-ASPH-positive labeling. Vehicle-treated cells show surface, cytoplasmic, and perinuclear FB50-ASPH immunoreactivity. SMI1182 treatment resulted in FB50-ASPH immunoreactivity localized along the inner surface of the plasma membrane or perinuclear rather than the cell surface. DOX treatment resulted in conspicuous bead-like fibrillar labeling of cell surface processes, particularly in larger tumor cells (see inset). BEZ treatment resulted in high percentages of pyknotic/shrunken cells with condensed immunostained nuclei and increased surface process and bleb FB50-ASPH immunoreactivity.

ELISA and immunocytochemical staining studies were used to compare the effects of SMI1182, DOX, and BEZ on ASPH immunoreactivity. Parallel studies examined immunoreactivity to cytoskeletal proteins for correlation with the cytomorphologic treatment effects. In CS1 cells, BEZ and SMI1182 significantly reduced A85G6-ASPH immunoreactivity (Figure 10A), whereas SMI1182 singularly increased FB50-ASPH (Humbug) (Figure 10B), GAPDH (Figure 10C), and β-Actin (Figure 10D) immunoreactivities relative to vehicle. In addition, vimentin immunoreactivity was elevated by each of the treatments relative to the control (Figure 10E). Immunocytochemical staining with the FB50-ASPH antibody revealed cell surface, cytoplasmic, perinuclear, and beaded fibrilla process labeling in vehicle-treated cultures (Figure 11-Left). SMI1182 treatment resulted in ASPH immunoreactivity tightly localized along the inner subjacent region of the plasma membrane or in the nucleus rather than on the outer membrane surfaces (Figure 11-Center Left). DOX treatment resulted in prominently fibrillar and beaded cell surfaces rather than cytoplasmic or perinuclear FB50-ASPH immunoreactivity (Figure 11-Center Right). BEZ treatment resulted in similar patterns of FB50-ASPH immunoreactivity as observed in the control CS1 cells, but with notably increased dense nuclear, short surface fibrillar, and surface bleb staining patterns (Figure 11-Right).

### 3.6. Cell Motility Studies

**Figure 12:**
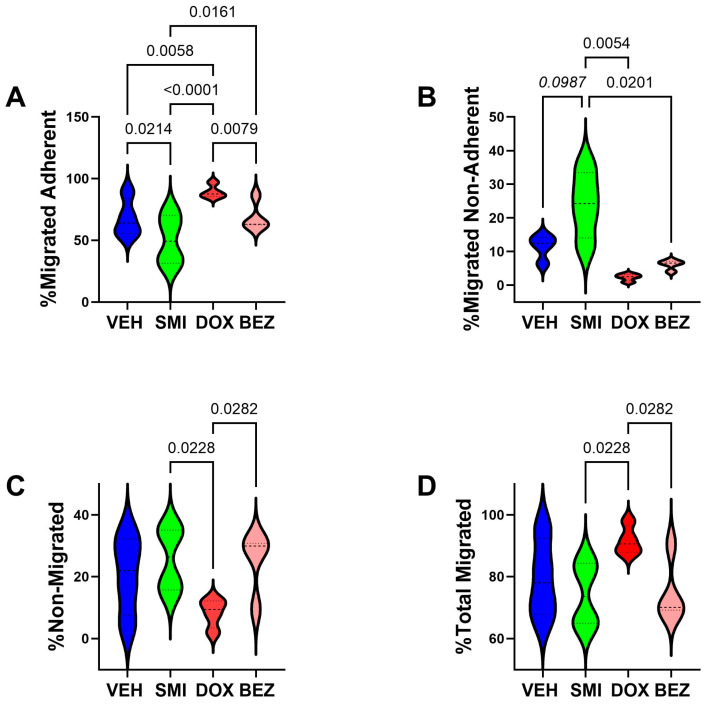
Effects of SMI1182, DOX, and BEZ on directional cell motility and adhesion. CS1 cells were treated for 48 h with vehicle (VEH), SMI1182 (SMI), DOX, or BEZ, then transferred to a Boyden chamber-type apparatus containing uncoated 8 µM pore diameter polycarbonate filters. Cell migration was allowed to proceed for 2 h in a standard cell culture incubator. The ATPLite luminescence assay was used to measure the motility index, i.e., the percentages of (**A**) motile adherent (migrated through the pores but still adherent to the undersurface of the membrane), (**B**) motile non-adherent (migrated to the bottom well of the chamber), (**C**) non-migrated (remaining on the upper membrane surface), and (**D**) total motile (motile adherent + motile non-adherent). Results were analyzed by ANOVA with post hoc multiple comparisons. Significant (*p* ≤ 0.05) and statistical trendwise (0.05 < *p* < 0.10) *p*-values are displayed. Trendwise effects have italic font.

The effects of 3.125 µM SMI1182, 0.05 µM DOX, and 2 µM BEZ on directional motility were evaluated in CS1 cells using the ATP-Lyte Motility assay with uncoated polycarbonate membranes. Two-way ANOVA revealed significant effects of treatment (F = 175.3; DFn, DFd: 3,48; *p* < 0.0001) and treatment × motility index interactions (F = 5.561; DFn, DFd: 9,48; *p* < 0.0001). The post hoc multiple comparisons tests demonstrated that SMI1182 reduced the percentages of migrated adherent and increased the migrated non-adherent percentages relative to vehicle, DOX, and BEZ (Figure 12A,B). DOX significantly increased the percentage of migrated adherent cells and the overall total percentage of migrated cells relative to SMI1182 and BEZ (Figure 12A,D), and it increased the percentage of migrated adherent cells relative to vehicle (Figure 12A). In contrast, BEZ did not significantly alter motility indices relative to any of the treatments, including vehicle (Figure 12A–D). In essence, the effects of SMI1182 and DOX were opposite from one another; SMI1182 reduced cell motility and adhesion, whereas DOX increased adherent cell motility and overall cell motility.

### 3.7. Molecular Correlates of SMI1182-, DOX-, and BEZ-Mediated Effects in CS Cells

**Figure 13:**
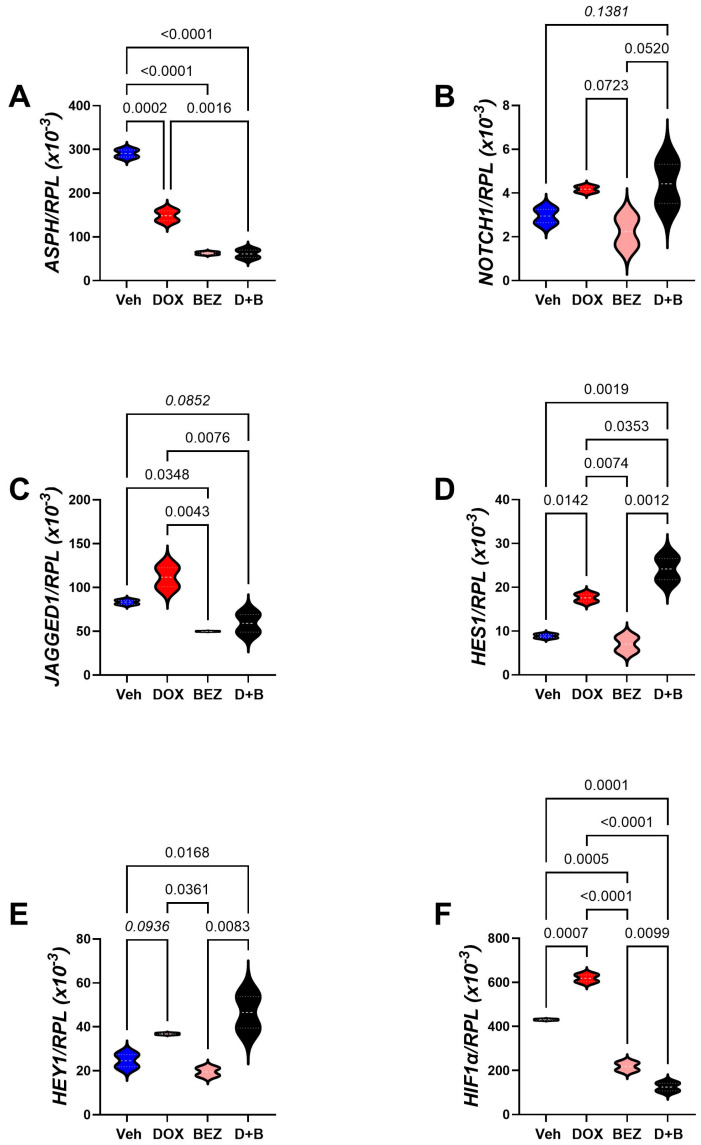
Molecular pathways of DOX-, BEZ-, and DOX+BEZ (D+B)-mediated CS1 cytotoxicity via ASPH and Notch networks. Cells (n = 4 cultures/group) were treated for 48h with Vehicle (Veh), DOX (0.05 µM), BEZ (2 µM), or D+B. (**A**) ASPH, (**B**) NOTCH1, (**C**) JAGGED1, (**D**) HES1, (**E**) HEY1, and (**F**) HIF-1α mRNA levels were measured using a multiplex bead-based RNA hybridization protocol with results normalized to Ribosomal Protein L13a (RPL) (see Table 3). Inter-group comparisons were made by one-way ANOVA with a post hoc repeated measures test. Significant (*p* ≤ 0.05) and statistical trendwise (0.05 < *p* < 0.10) *p*-values are displayed. Trendwise effects have italic font.

**Figure 14:**
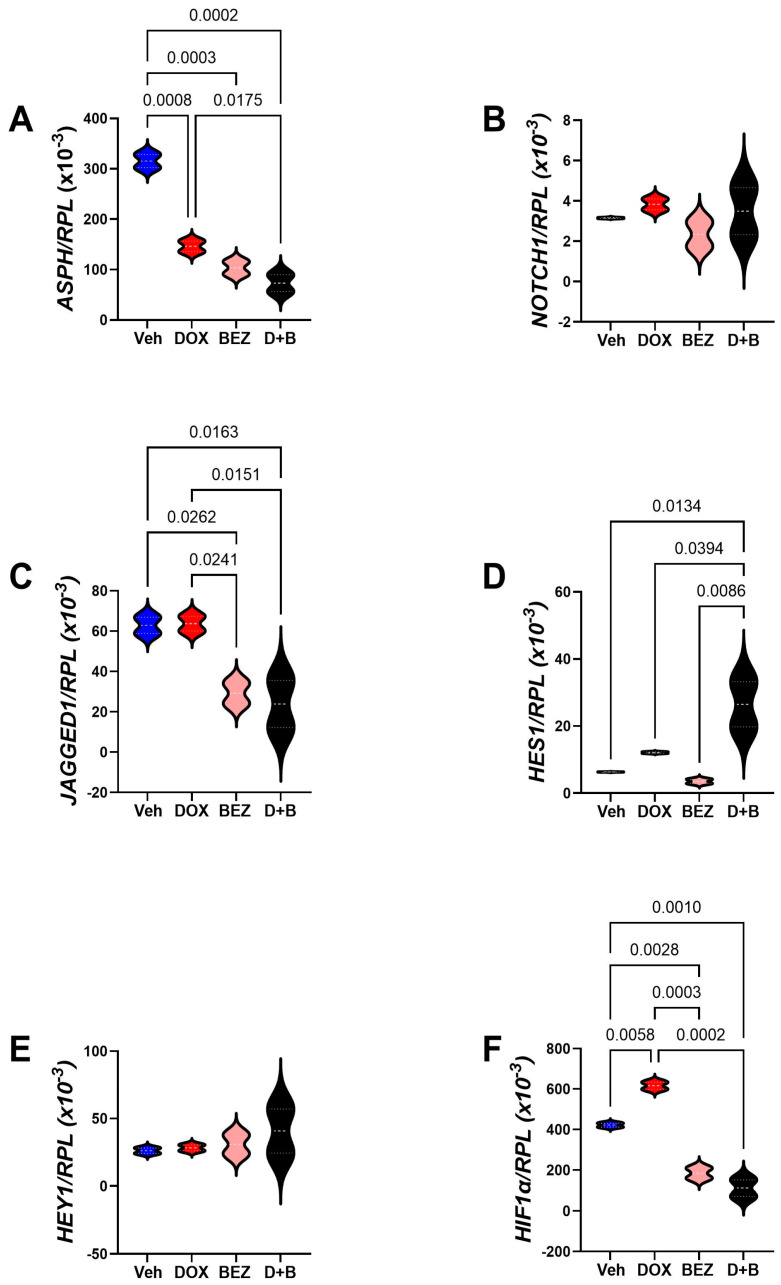
Molecular pathways of DOX-, BEZ-, and DOX+BEZ (D+B)-mediated CDS11 cytotoxicity via ASPH and Notch networks. Cells (n = 4 cultures/group) were treated for 48h with Vehicle (Veh), DOX (0.05 µM), BEZ (2 µM), or D+B. (**A**) ASPH, (**B**) NOTCH1, (**C**) JAGGED1, (**D**) HES1, (**E**) HEY1, and (**F**) HIF-1α mRNA levels were measured using a multiplex bead-based RNA hybridization protocol with results normalized to Ribosomal Protein L13a (RPL) (see Table 3). Inter-group comparisons were made by one-way ANOVA with a post hoc repeated measures test. Significant (*p* ≤ 0.05) and statistical trendwise (0.05 < *p* < 0.10) *p*-values are displayed. Trendwise effects have italic font.

**Figure 15:**
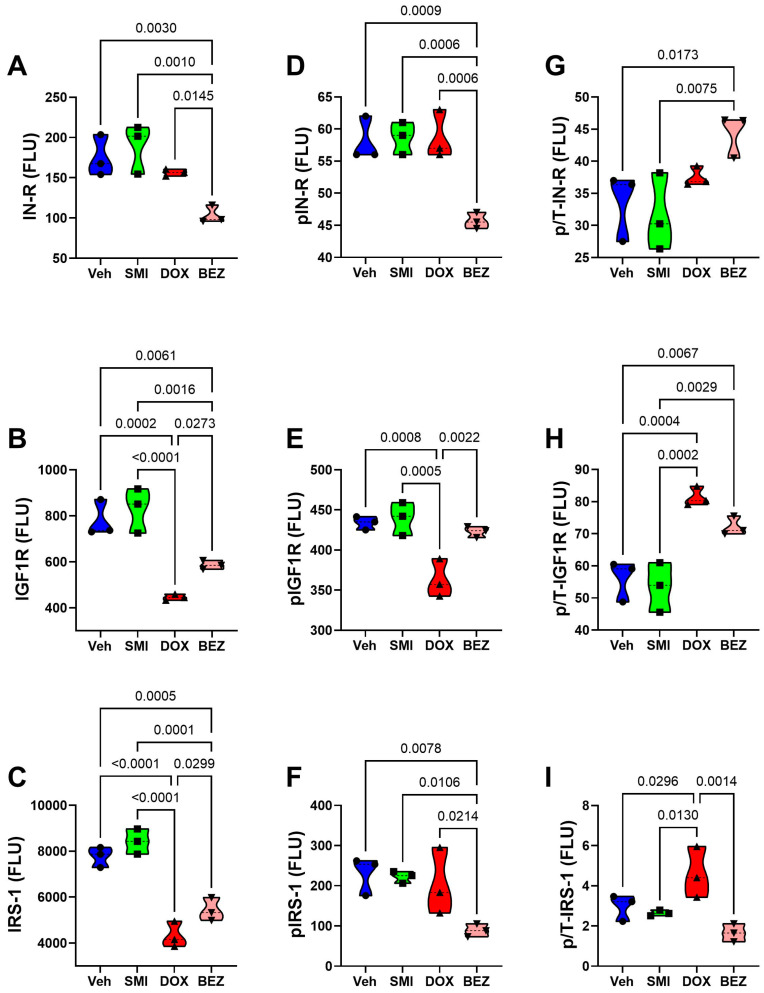
Effects of SMI1182 (SMI), DOX, and BEZ on the expression of upstream components of the Insulin/IGF1-Akt-mTOR pathway in CS1 cells. Commercial 11-plex magnetic bead-based ELISAs measured (**A**) Insulin receptor (IN-R), (**B**) IGF1R, (**C**) IRS-1, (**D**) pYpY1162/1163-IN-R, (**E**) pYpY1135/1136-IGF1R, and (**F**) pS636-IRS-1 and the calculated levels of relative phosphorylation of (**G**) p/T-IN-R, (**H**) p/T-IGF1R, and (**I**) p/T-IRS-1 in CS1 cells (n = 4/group) treated for 48 h with Veh, SMI, DOX, or BEZ. Values correspond to arbitrary fluorescent light units (FLU). Inter-group comparisons were made using two-way ANOVA tests (Table 5) with post -hoc Tukey tests. Software-generated *p*-values corresponding to significant differences (*p* ≤ 0.05) are shown in the panels.

**Figure 16:**
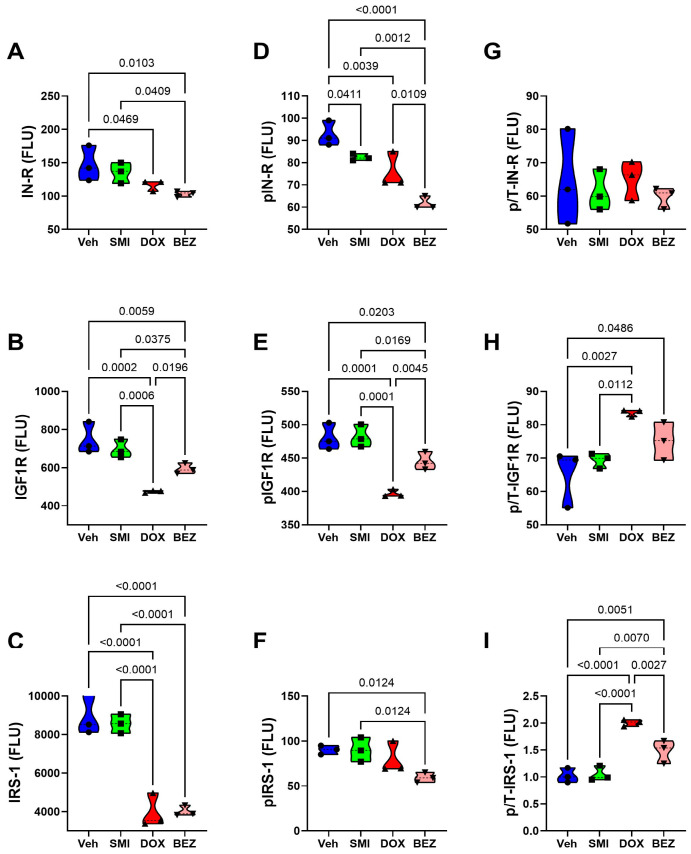
Effects of SMI1182 (SMI), DOX, and BEZ on the expression of upstream components of the Insulin/IGF1-Akt-mTOR pathway in CDS11 cells. Commercial 11-plex magnetic bead-based ELISAs measured (**A**) Insulin receptor (IN-R), (**B**) IGF1R, (**C**) IRS-1, (**D**) pYpY1162/1163-IN-R, (**E**) pYpY1135/1136-IGF1R, and (**F**) pS636-IRS-1 and the calculated levels of relative phosphorylation of (**G**) p/T-IN-R, (**H**) p/T-IGF1R, and (**I**) p/T-IRS-1 in CDS11 cells (n = 4/group) treated for 48h with Veh, SMI, DOX, or BEZ. Values correspond to arbitrary fluorescent light units (FLU). Inter-group comparisons were made using two-way ANOVA tests (Table 5) with post -hoc Tukey tests. Software-generated *p*-values corresponding to significant differences (*p* ≤ 0.05) are shown in the panels.

**Figure 17:**
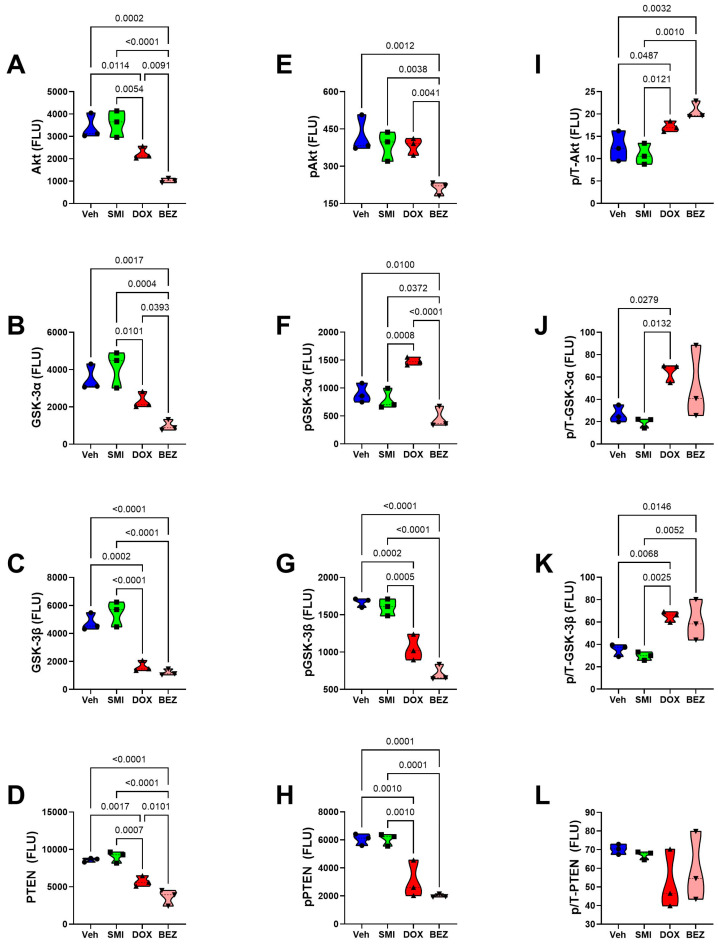
Effects of SMI1182 (SMI), DOX, and BEZ on the expression of mid-level components of the iInsulin/IGF1-Akt-mTOR pathway in CS1 cells. Magnetic bead-based protein and phosphoprotein 11-plex ELISAs measured frontal lobe levels of (**A**) Akt, (**B**) GSK-3α, (**C**) GSK-3β, (**D**) PTEN, (**E**) pS473-Akt, (**F**) pS21-GSK-3α, (**G**) pS9-GSK-3β, (**H**) pS380-PTEN, and the calculated levels of relative phosphorylation of (**I**) p/T-Akt, (**J**) p/T-GSK-3α, and (**K**) p/T-GSK-3β, and (**L**), and p/T-PTEN, (compared with total protein) in cells (n = 4/group) treated for 48 h with Veh, SMI, DOX, or BEZ. Graphed values correspond to arbitrary fluorescent light units (FLU). Inter-group comparisons were made using two-way ANOVA tests (Table 5) with post -hoc Tukey tests. Software-generated *p*-values corresponding to significant differences (*p* ≤ 0.05) are shown in the panels.

**Figure 18:**
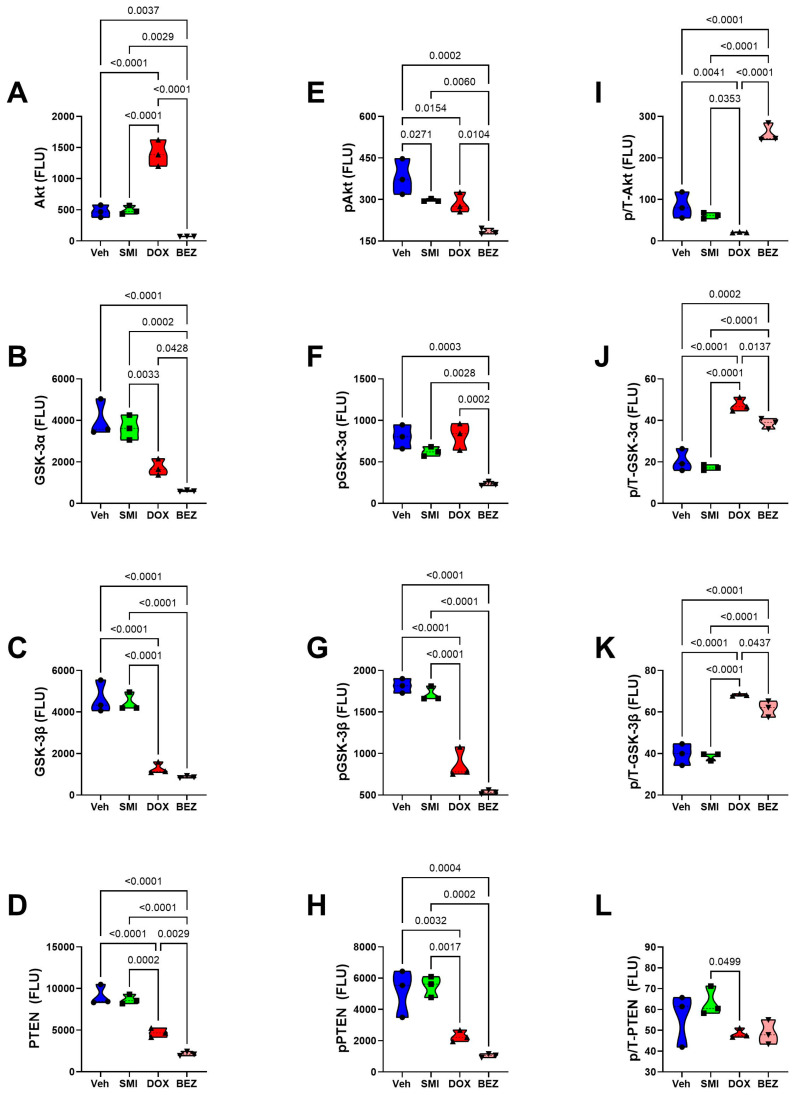
Effects of SMI1182 (SMI), DOX, and BEZ on the expression of mid-level components of the iInsulin/IGF1-Akt-mTOR pathway in CDS11 cells. Magnetic bead-based protein and phosphoprotein 11-plex ELISAs measured frontal lobe levels of (**A**) Akt, (**B**) GSK-3α, (**C**) GSK-3β, (**D**) PTEN, (**E**) pS473-Akt, (**F**) pS21-GSK-3α, (**G**) pS9-GSK-3β, (**H**) pS380-PTEN, and the calculated levels of relative phosphorylation of (**I**) p/T-Akt, (**J**) p/T-GSK-3α, and (**K**) p/T-GSK-3β, and (**L**), and p/T-PTEN, (compared with total protein) in cells (n = 4/group) treated for 48 h with Veh, SMI, DOX, or BEZ. Graphed values correspond to arbitrary fluorescent light units (FLU). Inter-group comparisons were made using two-way ANOVA tests (Table 5) with post -hoc Tukey tests. Software-generated *p*-values corresponding to significant differences (*p* ≤ 0.05) are shown in the panels.

**Figure 19:**
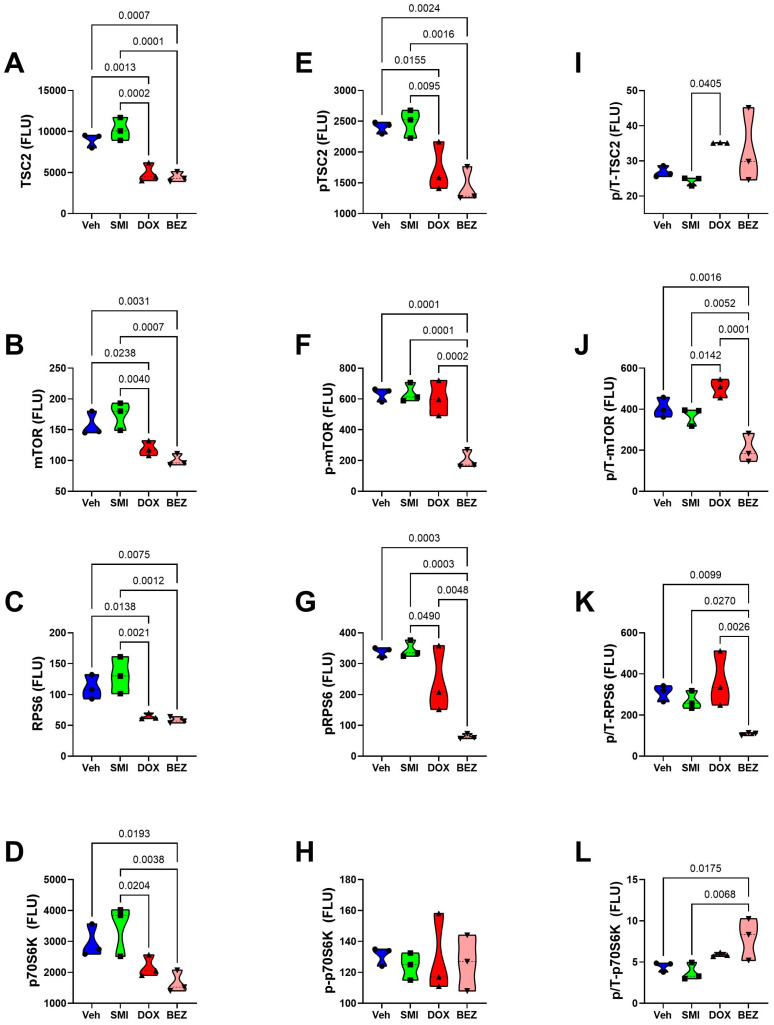
Effects of SMI1182 (SMI), DOX, and BEZ on the expression of downstream signaling through mTOR in CS1 cells. Magnetic bead-based protein and phosphoprotein 11-plex ELISAs measured immunoreactivity to (**A**) TSC2, (**B**) mTOR, (**C**) RPS6, (**D**) P70S6K, (**E**) pS939-TSC2, (**F**) pS2448-mTOR, (**G**) pS235/236-RPS6, (**H**) pT412-P70S6K, and the calculated relative levels of signaling molecule phosphorylation of (**I**) TSC, (**J**) mTOR, (**K**) RPS6, and (**L**) P70S6K in cells (n = 4 cultures/group) treated for 48 h with Veh, SMI, DOX, or BEZ. Graphed values correspond to arbitrary fluorescent light units (FLU). Inter-group comparisons were made by a two-way ANOVA (Table 5). Software-calculated *p*-values reflecting significant differences (*p* ≤ 0.05) are shown in the panels.

**Figure 20:**
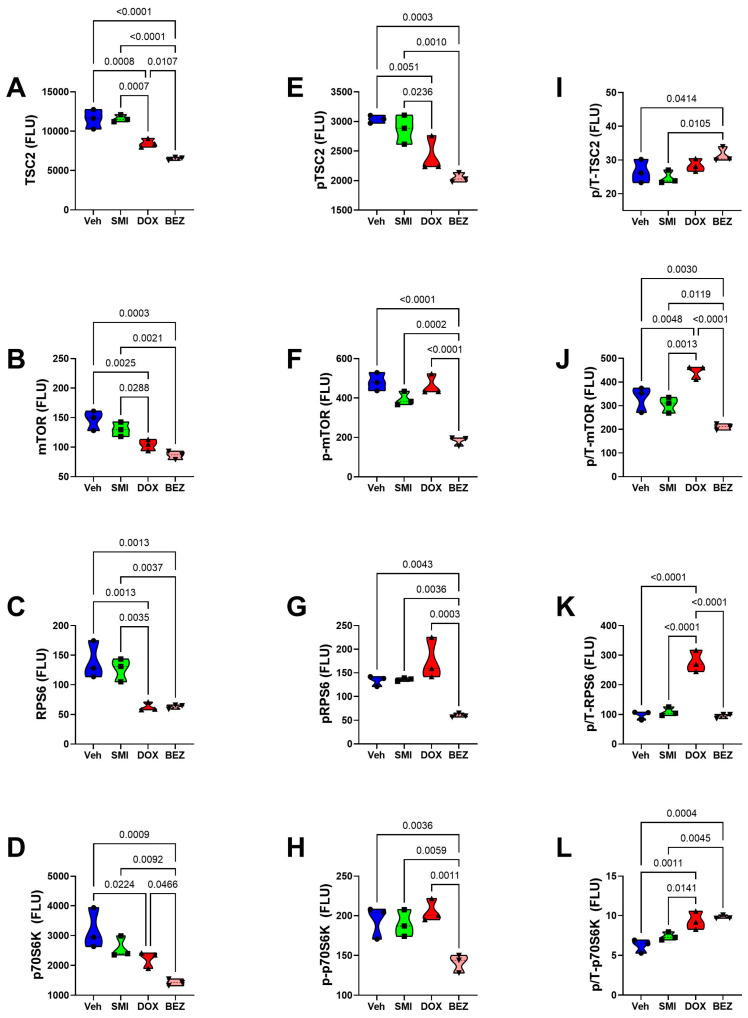
Effects of SMI1182 (SMI), DOX, and BEZ on the expression of downstream signaling through mTOR in CDS11 cells. Magnetic bead-based protein and phosphoprotein 11-plex ELISAs measured immunoreactivity to (**A**) TSC2, (**B**) mTOR, (**C**) RPS6, (**D**) P70S6K, (**E**) pS939-TSC2, (**F**) pS2448-mTOR, (**G**) pS235/236-RPS6, (**H**) pT412-P70S6K, and the calculated relative levels of signaling molecule phosphorylation of (**I**) TSC, (**J**) mTOR, (**K**) RPS6, and (**L**) P70S6K in cells (n = 4 cultures/group) treated for 48 h with Veh, SMI, DOX, or BEZ. Graphed values correspond to arbitrary fluorescent light units (FLU). Inter-group comparisons were made by a two-way ANOVA (Table 5). Software-calculated *p*-values reflecting significant differences (*p* ≤ 0.05) are shown in the panels.

The goal of these studies was to gain an understanding of the molecular distinctions between the high-grade CS1 and intermediate-grade CDS11 cells for correlation with malignant behavior and responsiveness to treatment. Such analyses could impact future strategies to improve the effectiveness of CS chemotherapy. Quantigene multiplex RNA hybridization assays were used to examine the effects of DOX, BEZ, and DOX+BEZ on the expression of the ASPH-Notch pathway (Figure 13 and Figure 14) and insulin/IGF signaling networks (Figures S3 and S4) that mediate cell growth, survival, migration, and invasion. In addition, we used multiplex bead-based ELISAs to measure total and phosphorylated molecules in the insulin/IGF-Akt-mTOR pathway in cells treated with vehicle, 10 µM SMI1182, 0.05 µM DOX, or 2 µM BEZ (Figure 15, Figure 16, Figure 17, Figure 18, Figure 19 and Figure 20). The treatment doses were based on the 50% inhibitory concentrations of the compounds in CS1/CDS11 cells.

**Table 3:** CS1 multiplex RNA hybridization assay ANOVA test results.

Gene Code	Gene Name	F-Ratio	*p*-Value
*ASPH*	Aspartyl-Asparaginyl-β-Hydroxylase	178.8	0.0001
*NOTCH1*	Notch 1	6.260	0.05
*JAGGED1*	Jagged 1	13.66	0.014
*HES1*	Hairy and enhancer of split-1	43.44	0.0016
*HEY1*	HES-related family bHLH transcription factor	9.259	0.0268
*HIF1α*	Hypoxia-Inducible Factor 1-alpha	242.2	<0.0001
*INS*	Insulin	1.234	N.S.
*IGF1*	Insulin-Like Growth Factor 1	1.336	N.S.
*IGF2*	Insulin-Like Growth Factor 2	2.577	N.S.
*INSR*	Insulin Receptor	2.637	N.S.
*IGF1R*	Insulin-Like Growth Factor 1 Receptor	3.164	N.S.
*IGF2R*	Insulin-Like Growth Factor 2 Receptor	9.891	0.0254
*IRS1*	Insulin Receptor Substrate, Type 1	16.01	0.0108
*IRS2*	Insulin Receptor Substrate, Type 2	0.667	N.S.
*IRS4*	Insulin Receptor Substrate, Type 4	0.487	N.S.

Molecular pathways of DOX-, BEZ-, and DOX+BEZ (D+B)-mediated CS1 cytotoxicity via insulin, IGF, and IRS pathways. The mRNA levels were measured with a multiplex bead-based RNA hybridization protocol, with results normalized to Ribosomal Protein L13a (RPL). Inter-group comparisons were made by one-way ANOVA. See Figure S4 for the results of the Tukey post hoc repeated measures tests of all inter-group comparisons. n = 4 cultures/group. Significant (*p* ≤ 0.05) *p*-values are displayed. N.S. = not significant.

*ASPH-NOTCH* Panel: Comparisons were made with cells treated with DOX, BEZ, or DOX+BEZ (D+B). SMI1182 treatment effects were not studied because preliminary studies failed to demonstrate any changes in mRNA expression. One-way ANOVA tests detected significant inter-group differences with respect to *ASPH*, *NOTCH1*, *JAGGED1*, HES1, HEY1, and HIF-1α in CS1 cells (Table 3), and ASPH, *JAGGED1*, *HES1*, and *HIF-1α* for CDS11 cells (Table 4). Post hoc tests demonstrated that DOX treatment of CS1 cells significantly reduced ASPH (Figure 13A) but either significantly or trendwise increased *NOTCH 1* (Figure 13B)*, HES1* (Figure 13D)*, HEY1* (Figure 13E), and *HIF1α* (Figure 13F) relative to vehicle. BEZ significantly reduced *ASPH* (Figure 13A), *JAGGED1* (Figure 13C), and *HIF-1a* (Figure 13F). Dual DOX+BEZ mimicked the inhibitory effects of BEZ monotherapy on *ASPH*, *JAGGED1*, and *HIF1a*, as well as DOX’s upregulated expression of *NOTCH1*, *HES1*, and *HEY1*. In CDS11 cells, DOX significantly inhibited *ASPH* (Figure 14A) but had no significant effects on *NOTCH1* (Figure 14B), *JAGGED1* (Figure 14C), *HES1* (Figure 14D), or *HEY1* (Figure 14E), and increased *HIF1α* (Figure 14F) relative to vehicle. BEZ significantly reduced *ASPH*, *JAGGED1,* and *HIF1α* but not *NOTCH1*, *HES1*, *or HEY1*. Dual DOX+BEZ treatments significantly reduced *ASPH*, *JAGGED1*, and *HIF1α* but increased *HES1* relative to the other treatments in CDS11 cells.

*INSULIN/IGF/IRS* pathway mRNA transcripts were measured with a custom QuantiPlex assay, and the results were normalized to *RPL13a*. One-way ANOVA tests detected significant treatment effects on *IGF2R* and *IRS1* in CS1 cells (Table 3) and a significant effect on *IRS1* and a statistical trendwise effect on *INSULIN* mRNA expression in CDS11 cells (Table 4). For CS1 cells (Figure S4), post hoc tests showed significant DOX-mediated increases in *IGF1R* (Figure S4E) and *IGF2R* (Figure S4F) and BEZ- and DOX+BEZ-mediated increases in *IGF2R* (Figure S4F), as well as reductions in *IRS1* (Figure S4G). For CDS11 cells, the post hoc tests demonstrated significantly lower *INSULIN* (Figure S5A) and *IRS1* (Figure S5G) mRNA levels with BEZ or DOX+BEZ, and increased *IGF2R* associated with BEZ (Figure S5F).

### 3.8. Akt-mTOR Pathway Analysis

To examine the effects of SMI1182, DOX, and BEZ on insulin/IGF/IRS1 signaling through Akt pathways to mTOR, we measured total and phosphorylated proteins using multiplex bead-based ELISA platforms. In addition, to assess the effects on relative levels of phosphorylation, we calculated the ratios of phosphorylated to total protein immunoreactivity. Results were analyzed by ANOVA with repeated measures post hoc Tukey tests.

CS1 Cells: SMI1182 had no statistically significant or trendwise effects on the expression of upstream (Insulin-R, IGF1R, IRS1) (Figure 15), intermediary (Akt, GSK-3α, GSK-3β, PTEN) (Figure 17), or downstream (TSC2, mTOR, RPS6, p70S6K) (Figure 19) proteins, phosphoproteins, or the relative levels of protein phosphorylation (p/T) assessed in the panel. DOX significantly reduced the levels of both the IGF1R (Figure 15B), tyrosine phosphorylated IGF1R (Figure 15E), IRS-1 (Figure 15C), Akt (Figure 17A), GSK-3β (Figure 17C), p-GSK-3β (Figure 17G), PTEN (Figure 17D), p-PTEN (Figure 17H), TSC2 (Figure 19A), p-TSCE (Figure 19E), mTOR (Figure 19B), and RPS6 (Figure 19C), and increased p-GSK-3α (Figure 17F). The discordant or unbalanced inhibition of total versus phosphoprotein expression resulted in significant increases in relative phosphorylation of IGF1R (Figure 15H), IRS-1 (Figure 15I), Akt (Figure 17I), and GSK-3α (Figure 17J) in the DOX versus vehicle-treated CS1 cells. BEZ significantly reduced the levels of all proteins and phosphoproteins except for p-IGF1R and p-p70S6K, indicating that the dominant effect of BEZ was to broadly inhibit the expression of nearly all critical signaling molecules in the Akt-mTOR pathway in CS1 cells.

**Table 5:** CS grade and treatment effects on insulin/IGF1/IRS-Akt-mTOR signaling.

	Treatment	*p*-Value	CS Grade	*p*-Value	Treatment × CS Grade	*p*-Value
IN-R	13.26	0.0001	16.07	0.001	2.285	N.S.
p-IN-R	29.07	<0.0001	175.5	<0.0001	6.212	0.005
IGF1R	39.36	<0.0001	1.979	N.S.	2.400	N.S.
p-IGF1R	33.27	<0.0001	31.05	<0.0001	0.703	N.S.
IRS1	81.54	<0.0001	0.25	N.S.	4.209	0.0225
p-IRS1	7.535	0.0023	52.91	<0.0001	3.132	*0.054*
Akt	28.64	<0.0001	224.2	<0.0001	22.0	<0.0001
p-Akt	21.07	<0.0001	11.58	0.0036	0.829	N.S.
GSK-3α	34.43	<0.0001	0.863	N.S.	1.05	N.S.
p-GSK-3α	34.54	<0.0001	25.68	<0.0001	5.146	0.01
GSK-3β	90.35	<0.0001	4.49	0.05	0.785	N.S.
p-GSK-3β	132.8	<0.0001	0.284	N.S.	3.563	0.038
PTEN	91.46	<0.0001	3.718	*0.0718*	1.89	N.S.
p-PTEN	43.21	<0.0001	6.12	0.0249	0.073	N.S.
TSC	53.03	<0.0001	43.21	<0.0001	1.602	N.S.
p-TSC	23.81	<0.0001	35.48	<0.0001	0.447	N.S.
mTOR	24.57	<0.0001	12.28	0.0029	1.68	N.S.
p-mTOR	49.11	<0.0001	31.42	<0.0001	3.459	0.041
RPS6	23.97	<0.0001	0.764	N.S.	0.909	N.S.
p-RPS6	23.48	<0.0001	47.73	<0.0001	8.774	0.001
P70S6K	13.53	0.0001	1.191	N.S.	1.475	N.S.
p-P70S6K	5.049	0.012	68.16	<0.0001	4.346	0.02

Two-way ANOVA test results reflecting CS grade (CS1-Grade 3 versus CDS11 cells-Grade 2), treatment, and CS grade × treatment interactive effects on the expression of signaling and phosphorylated signaling molecules (^pYpY1162/1163^-Insulin R, ^pYpY1162/1163^-IGF1R, ^pS636^-IRS-1, ^pS473^-Akt, ^pS21^-GSK-3α, ^pS9^-GSK-β, 3 ^pS380^-PTEN, ^pS939^-TSC2, ^pS2448^-mTOR, ^pS235/S236^-RPS6, ^pT412^-P70S6K) in the Akt/mTOR pathway. Significant differences (*p* ≤ 0.05) and statistical trend (0.05 < *p* < 0.10; italic) results are displayed. N.S. = not significant. n = 4 cultures/group. See Figure 15, Figure 16, Figure 17, Figure 18, Figure 19 and Figure 20 for corresponding graphs and post hoc test results. Abbreviations: R = receptor; IGF = insulin-like growth factor; IRS = insulin receptor substrate; GSK = glycogen synthase kinase; PTEN = phosphatase and tensin homolog; TSC = tuberous sclerosis complex 2; mTOR = mechanistic target of rapamycin; RPS6 = Ribosomal Protein S6; P70S6K = 70 kDa ribosomal protein S6 kinase.

CDS11 Cells: As observed in CS1 cells, SMI1182 monotherapy had no significant or trendwise effects on any of the signaling proteins or phosphoproteins analyzed with the multiplex panels, except for pAkt, which was significantly reduced relative to the vehicle control (Figure 16, Figure 18, and Figure 20). DOX significantly reduced Insulin R (Figure 16A), IGF1R (Figure 16B), IRS1 (Figure 16C), GSK-3β (Figure 18C), PTEN (Figure 18D), TSC2 (Figure 20A), mTOR (Figure 20B), RPS6 (Figure 20C), and p70S6K (Figure 20D), with limited concordant inhibitory effects on corresponding phosphoproteins, including p-Insulin R (Figure 14D), p-IGF1R (Figure 16E), p-GSK-3β (Figure 18G), p-PTEN (Figure 18H), and pTSC2 (Figure 20E). In addition, DOX had discordant inhibitory effects on p-Akt (Figure 18E). BEZ had broad inhibitory effects on all protein and phosphoprotein signaling molecules in the panel (Figure 16, Figure 18, and Figure 20).

### 3.9. Comparative Effects of Treatment in Relation to CS Grade

Two-way ANOVA tests compared the effects of CS grade, treatment, and CS grade × treatment interactions on the expression of insulin/IGF/IRS-Akt-mTOR pathway proteins and phosphoproteins. SMI1182 results were excluded from these analyses because we did not detect any significant or statistical trendwise effects relative to the vehicle. Treatment significantly modulated the expression of all 22 signaling proteins and phosphoproteins (Table 5). CS grade (III-CS1; II-CDS11) significantly modulated the expression of 5 of the 11 (45.5%) signaling molecules and 10 of 11 (90.9%) phosphoproteins. Treatment × CS grade interactive effects were observed for two (18.1%) proteins and seven (63.6%) phosphoproteins. Importantly, these results indicate substantial effects of treatment modality and CS grade, as well as interactive effects of treatment and CS grade.

## 4. Discussion

The goals of this study were to characterize the expression and regulation of ASPH in moderate- versus high-grade human conventional CS cells and examine the differential effects of treatments designed to target molecular pathways that mediate ASPH’s expression and function. ASPH stands out as an important therapeutic target in malignancy [26,78] due to its high levels of expression on cancer cell surfaces and very low or absent expression in most normal cells [34]. Identifying new therapeutic targets for CS is a priority set by the American Academy of Orthopaedic Surgeons. To this end, we extended our investigations beyond DOX, one of the standard chemotherapeutic agents used to treat post-surgical recurrent CS [79], by also examining the effects of SMI1182, a small molecule inhibitor of ASPH, and BEZ235, a potent inhibitor of PI3K/mTOR [80,81]. BEZ235 was studied because PI3K/mTOR signaling plays a critical role in sarcoma progression [10,82,83], and BEZ235’s inhibitory effects on cancer cell growth are independent of EGFR status [84].

This study utilized two established human conventional CS cell lines regarded as histologic Grade 3 (CS1) or Grade 2 (CDS11). Efforts were made to delineate their similarities and differences in relation to ASPH expression and function, as well as their sensitivity to SMI1182, DOX, BEZ, and combination treatment. Corresponding with previous studies, higher levels of ASPH were detected in CS1 compared with CDS11 cells. CDS11 cells were further distinguished from CS1 by their higher level of Humbug (FB50-ASPH), which has been reported for malignancies such as those of gastrointestinal lineage [69,73,75]. Despite higher levels of ASPH in CS1 cells, the motility indices were like those of CDS11 cells, suggesting that factors other than ASPH levels govern CS motility and invasion potential. For example, the increased Humbug expression in CDS11 cells may be significant in relation to its Ca2+ binding effects in the ER [85]. Other findings reflective of CS1’s higher tumor grade and more aggressive behavior compared with CDS11 cells include the significantly higher mRNA levels of *INSULINR*, *IGF2R*, *IRS1*, *NOTCH1*, *JAGGED1*, *HES1*, and *HIF1α*, which function to promote tumor cell growth and survival.

Various experiments compared the effects of treatment in CS1 and CDS11 cells to gain a better understanding of how therapeutic approaches can be optimized based on tumor grade. MTT and viability assays demonstrated that CS1 cells were relatively resistant to DOX but sensitive to BEZ, whereas CDS11 cells were sensitive to DOX but relatively resistant to BEZ. However, with a combination of DOX+BEZ treatment, both CS1 and CDS11 cells were effectively killed, indicating the importance of multi-targeting therapeutic approaches for both high- and intermediate-grade CS. Surprisingly, the impact of SMI1182 was inconsistent, as it primarily enhanced the BEZ killing of CDS11 cells and also exhibited small but significant anti-tumor effects in DOX-treated CS1 cells, but it produced cytoprotection in DOX-treated CDS11 cells. Notably, these conclusions were based on H33342 data, reflecting cell loss, since corresponding increases in MTT activity in CS1 cells co-administered BEZ and DOX or DOX+SMI1182 were likely related to oxidative stress. The SMI1182 dose–response and co-treatment studies confirmed that SMI1182 was largely ineffective as a monotherapy but suitable as an adjuvant for enhancing the chemotherapeutic effects of DOX or BEX. Correspondingly, preliminary studies suggest that while cytotoxic effects of SMI1182 monotherapy occur at doses above 10 µM CS1 and CDS11 cells, synergistic cytotoxicity with 0.5 µM DOX may occur with as little as 3.125 µM SMI1182.

Altogether, these findings highlight greater DOX monotherapy resistance in CS1 versus CDS11 cells and greater BEZ monotherapy resistance in CDS11 versus CS1 cells. However, the studies showed significant and largely similar benefits of DOX+BEZ or DOX+BEZ+SMI combination therapy for anti-tumor cell effects in high-grade (CS1) and intermediate-grade (CDS11) CS. The concerns revealed by these studies were that (1) although the SMI1182 ASPH inhibitor enhanced the DOX killing of CS1 cells, in CDS11 cells, it had opposite effects when administered in combination with DOX; (2) BEZ or BEZ+SMI combined with DOX effectively killed CS1 cells to greater extents than DOX only—this was not the case for CDS11 cells; (3) the BEZ killing of CS1 cells was incrementally increased by the addition of SMI, DOX, or SMI+DOX but strikingly increased by the co-treatment of CDS11 cells with BEZ+ SMI, DOX, or SMI+DOX. These differential responses to monotherapy and combination therapy underscore the importance of considering CS grade as a key variable in regulating the effectiveness of novel combination chemotherapy protocols. Correspondingly, several studies have demonstrated at least additive effects of dual treatment with BEZ235 and DOX for enhancing cancer cell killing and reducing tumor cell growth [86,87,88].

The cytomorphological effects of treatment reflected the impact of treatment on cell viability and cytotoxicity. Correspondingly, the chamber slide cultures showed that SMI1182 treatment yielded similar growth patterns and cellular morphology as with vehicle treatment, whereas the cytotoxic effects of BEZ or DOX were manifested by conspicuous cell loss, apoptosis, and cellular necrosis. Cytospin preparations provided better opportunities to assess treatment effects on cell shape and appreciate the degree to which BEZ induced cell cycle arrest and apoptosis. BEZ-associated surface membrane blebbing, i.e., irregular bulging of the plasma membrane, likely reflected the decoupling of the cytoskeleton from the plasma membrane. Physiologically, the response could occur during cell migration, such as cancer cell metastasis [89], or with apoptosis [90]. The prominent cell shrinkage with nuclear condensation associated with DOX or BEZ, with or without SMI1182, is consistent with known mechanisms of DOX- [91] and BEZ-mediated [84,92] cancer cell killing, including necrosis, cytotoxicity, autophagy, and apoptosis. In contrast, the cell swelling, pallor, nuclear loss, and fragmentation noted more in SMI1182-treated cells likely represented apoptosis as a prominent mechanism of cell death linked to Notch pathway inhibition [93,94,95].

Previous studies have shown that ASPH plays functional roles in cell motility and invasion through its catalytic activity, which hydroxylates Asp and Asn residues in the EGF-like domains of Notch and Jagged (Ince, 2000 #52). SMI’s targeting ASPH’s catalytic domain was shown to reduce malignant neoplastic cell motility by inhibiting the expression of Notch transcription factors rather than *ASPH*, *NOTCH*, or *JAGGED* [36,38]. Herein, the SMI1182 treatment of CS cells reduced ASPH-A85G6 expression, ASPH cell surface immunoreactivity, and the percentages of migrated adherent cells. BEZ also reduced ASPH immunoreactivity and its cell surface localization but did not impact motility relative to the vehicle, most likely due to its failure to reduce *NOTCH*, *HES1*, and *HEY1*. Apart from its selective inhibition of *ASPH* mRNA, DOX exhibited anti-therapeutic effects, including enhanced migration, Notch pathway signaling, and ASPH surface fibrillary staining in CS1 cells. Reduced surface ASPH immunoreactivity in SMI1182-treated cells may have been instrumental in impairing the hydroxylation of EGF-like domains of Notch and Jagged and thereby impairing cell motility, whereas increased surface fibrillar localization of ASPH immunoreactivity associated with DOX may have contributed to the increased CS cell motility.

The findings with respect to SMI1182 suggest that the inhibition of ASPH’s catalytic activity, coupled with reduced ASPH surface localization, accounts for its inhibitory effects on cell motility. The additional findings that DOX-associated increases in *IGF1R*, *IGF2R*, and *IRS1* in CS1 cells, and BEZ and DOX+BEZ reductions in *IRS1* expression in CS1 and CDS11 cells, are significant because increased signaling through insulin/IGF/IRS corresponds with the observed increases in cell motility. Further studies designed to better understand the mechanistic differential treatment responses of CS1 versus CDS11 cells revealed that DOX treatment of CS1 cells mainly enhanced the expression of Notch pathway signaling molecules, whereas BEZ treatment inhibited the key mRNAs, including JAGGED, HES1, and HIF1α, which have important roles in cell migration and invasiveness [96,97]. The largely stimulatory effects of DOX on Notch pathway signaling in CS1 cells versus its limited effects in CDS11 cells may account for high-grade CS resistance to DOX monotherapy. The BEZ-mediated reduction in *HES1* mRNA suggests that BEZ can have inhibitory effects on Notch with attendant reductions in critical transcription factors [30,31,38]. These findings are consistent with previous reports that BEZ or a related PI3K/mTOR inhibitor can have significant anti-cancer/therapeutic effects in malignancies, including high-grade CS [98,99,100], and that BEZ235 can enhance the cytotoxic effects of DOX in malignancies [86,88], in part due to the BEZ-mediated increased accumulation of DOX [87]. However, there are caveats signaled by the finding that dual treatment with BEZ235 can also worsen outcomes by further increasing Notch pathway activity, as reported previously [101]. Similarly, the co-administration of DOX with cisplatin or knockdown of *Notch1* in osteosarcoma reduced the cytotoxic effects of DOX via the unanticipated upregulation of Notch1 and related target genes [102].

The final approach for characterizing molecular mediators of tumor cell responsiveness based on CS grade was to examine the effects of SMI1182, DOX, and BEZ on insulin/IGF/IRS signaling downstream through mTOR networks using multiplex ELISA platforms. DOX and BEZ significantly reduced the expression of most upstream (Insulin-R, IGF1R, and IRS1), intermediate (Akt, GSK-3, and PTEN), and downstream (TSC2, mTOR, RPS6, and p70S6K) signaling mediators in both CS1 and CDS11 cells. The few exceptions were that DOX had no significant effect on Insulin-R, GSK-3α, or p70S6K in CS1 cells, and it upregulated Akt in CDS11 cells. It is noteworthy that the reduced levels of IRS1 protein were concordant with the effects of DOX and BEZ on IRS1 mRNA, whereas the downward shifts in Insulin-R and IGF1R were detected by multiplex ELISA and not mRNA analysis, suggesting post-transcription mechanisms lead to the increased degradation of signaling molecules. The broadly parallel reductions in phosphoproteins correlated with the downregulated expression of specific signaling molecules and, therefore, were unlikely to have been caused by the selective disruption of kinase activities. In contrast, SMI1182 treatment had no significant effects on the expression levels of signaling molecules or their corresponding phosphoproteins, indicating that its anti-cancer effects were produced by alternative indirect mechanisms linked to ASPH’s functions. In terms of distinguishing treatment responses between high-grade and intermediate-grade CS, the multiplex assays demonstrated DOX inhibition of Insulin-R and p-Insulin-R in CDS11 but not CS1 cells, and increased relative levels of GSK-3b phosphorylation (silencing) in CS1 but not CDS11 cells, suggesting that the failure of DOX to inhibit insulin signaling in CS1 cells, combined with the suppression of GSK-3β activity, contributes to DOX chemotherapy resistance in CS1 cells. On the other hand, the differential inhibitory effects of BEZ on IGF1R and p70S6K phosphorylation in CDS11 but not CS1 cells may have contributed to its sharp dose-dependent reductions in CDS11 cell viability.

## 5. Conclusions

This study characterized ASPH expression in human CS cells and demonstrated that high-grade CS cells expressed higher levels of ASPH, Notch pathway genes, and selected insulin/IGF/IRS pathway-related genes compared with intermediate-grade CS. The identified differences could account for the more aggressive behavior of high-grade versus intermediate-grade CS.

SMI1182, DOX, and BEZ independently inhibited CS cell viability and increased cytotoxicity in a dose-dependent manner. However, combination therapy with SMI1182+BEZ, SMI1182+DOX, or BEZ+DOX mainly had additive effects on CS cell cytotoxicity/killing compared with monotherapy.

The therapeutic effects of SMI1182, DOX, and BEZ were mediated in part by the inhibition of ASPH expression, Notch pathway genes, and signaling through the insulin/IGF/IRS-Akt-mTOR pathways. The greater inhibitory effects of BEZ versus DOX or SMI1182 were associated with broader impairments in signaling molecule expression, including in CDS11 (intermediate grade) versus CS1 (high grade) cells.

The SMI1182 small molecule inhibitor of ASPH functioned more effectively as a cofactor or adjuvant agent with DOX or BEZ for killing CS cells rather than as a monotherapy.

Combination therapy with DOX or BEZ plus SMI1182 could improve clinical outcomes from CS.

## Figures and Tables

**Table 1:** Antibodies used: characteristics, sources, and concentrations.

Antibody Name *	Antibody Type	Source	Catalog #	Concentration
A85G6-ASPH)	Mouse Monoclonal	Liver Research Center, Brown University Health, RI USA	A85G6	1.3 µg/mL
FB50-ASPH)	Mouse Monoclonal	Liver Research Center, Brown University Health, RI USA	FB50	0.845 µg/mL
GAPDH	Mouse Monoclonal	Santa Cruz Biotechnology	sc-365062	0.067 µg/ml
β-Actin	Mouse Monoclonal	Santa Cruz Biotechnology	sc-47778	0.2 µg/mL
Vimentin	Mouse Monoclonal	RV-202; Abcam	ab8978	2.5 µg/mL
RPLPO	Mouse Monoclonal	Santa Cruz Biotechnology	sc-293260	0.1 µg/mL
HRP-conjugated secondary	Goat Polyclonal	Thermo Fisher Scientific;	Anti-mouse-31430Anti-rabbit-31461	0.04 µg/mL

* Abbreviations: ASPH = aspartyl-asparaginyl-β-hydroxylase; GAPDH = glyceraldehyde-3-phosphate dehydrogenase; HRP = horseradish peroxidase; RPLPO = large acidic ribonuclear protein. **#** Has been defined to reflect abbreviations in the table.

**Table 2:** The 11-Plex Akt-mTOR pathway (proteins and phosphoproteins).

Akt/mTOR Pathway Molecules	Protein Abbreviation	Phosphoprotein
Insulin Receptor	Insulin-R	^pYpY1162/1163^-Insulin R
Insulin-Like Growth Factor Receptor Type 1	IGF-1R	^pYpY1135/1136^-IGF-1R
Insulin Receptor Substrate, Type 1	IRS1	^pS636^-IRS-1
Akt (Protein Kinase B)	Akt	^pS473^-Akt
Phosphatase and tensin homolog	PTEN	^pS380^-PTEN
Glycogen Synthase Kinase 3α	GSK-3α	^pS21^-GSK3α
Glycogen Synthase Kinase 3β	GSK-3β	^pS9^-GSK3β
Tuberous Sclerosis Complex 2	TSC2	^pS939^-TSC2
Mechanistic Target of Rapamycin	mTOR	^pS2448^-mTOR
p70 Ribosomal S6 kinase	P70S6K	^pT412^-p70S6K
Ribosomal Protein S6	RPS6	^pS235/S236^-RPS6

**Table 4:** CDS11 multiplex RNA hybridization assay ANOVA test results.

Gene Code	Gene Name	F-Ratio	*p*-Value
*ASPH*	Aspartyl-Asparaginyl-β-Hydroxylase	66.64	0.0007
*NOTCH1*	Notch 1	0.8998	N.S.
*JAG1*	Jagged 1	9.533	0.0270
*HES1*	Hairy and enhancer of split-1	9.187	0.0288
*HEY1*	HES-related family bHLH transcription factor	0.5119	N.S.
*HIF1α*	Hypoxia-Inducible Factor 1-alpha	80.51	0.0005
*INS*	Insulin	*4.845*	*0.0808*
*IGF1*	Insulin-Like Growth Factor 1	0.5891	N.S.
*IGF2*	Insulin-Like Growth Factor 2	0.8381	N.S.
*INSR*	Insulin Receptor	0.4804	N.S.
*IGF1R*	Insulin-Like Growth Factor 1 Receptor	1.100	N.S.
*IGF2R*	Insulin-Like Growth Factor 2 Receptor	2.613	N.S.
*IRS1*	Insulin Receptor Substrate, Type 1	13.75	0.0142
*IRS2*	Insulin Receptor Substrate, Type 2	1.245	N.S.
*IRS4*	Insulin Receptor Substrate, Type 4	0.4039	N.S.

Molecular pathways of DOX-, BEZ-, and DOX+BEZ (D+B)-mediated CDS11 cytotoxicity via insulin, IGF, and IRS pathways. The mRNA levels were measured with a multiplex bead-based RNA hybridization protocol, with results normalized to Ribosomal Protein L13a (RPL). Inter-group comparisons were made by one-way ANOVA. See Figure S5 for the results of Tukey post hoc repeated measures tests for all inter-group comparisons. n = 4 cultures/group. Significant (*p* ≤ 0.05) and statistical trendwise (0.05 < *p* < 0.10) (italics) *p*-values are displayed. N.S. = not significant.

## Data Availability

Data will be made available upon reasonable request from the corresponding author.

## Supplementary Materials

The following supporting information can be downloaded at: https://www.mdpi.com/article/10.3390/cancers17101671/s1, Figure S1: Directional cell motility and adhesion in CS1 and CDS11 cells; Figure S2: Directional cell motility and adhesion in CS1 and CDS11 cells; Figure S3: Molecular pathways of DOX-, BEZ-, and DOX+BEZ (D+B)-mediated CS1 cytotoxicity via insulin, IGF, and IRS pathways; Figure S4: Molecular pathways of DOX-, BEZ-, and DOX+BEZ (D+B)-mediated CDS11 cytotoxicity via insulin, IGF, and IRS pathways. Figure S5: Molecular Pathways of DOX-, BEZ-, and DOX+BEZ (D+B)-Mediated CDS11 Cytotoxicity Via Insulin, IGF, and IRS Pathways.

## Abbreviations

The following abbreviations are used in this manuscript:

**Abbreviation**	**Definition**
Akt	Akt (Protein Kinase B)
ASPH	Aspartyl-asparaginyl-β-hydroxylase
AUC	Area-under-curve
BEZ	BEZ235; combination of everolimus and the imidazoquinoline derivative
CS	Chondrosarcoma
DMSO	Dimethyl sulfoxide
DOX	Doxorubicin
EDTA	Ethylenediaminetetraacetic acid
EIA	Enzyme immunoassay
ELISA	Enzyme-linked immunosorbent assay
GAPDH	Glyceraldehyde-3-phosphate dehydrogenase
GSK-3a	Glycogen Synthase Kinase 3a
GSK-3β	Glycogen Synthase Kinase 3β
HES1	Hairy and enhancer of split-1
HEY1	HES-related family bHLH transcription factor
HIF1α	Hypoxia-Inducible Factor 1-alpha
HRP	Horseradish peroxidase
IGF1	Insulin-Like Growth Factor 1
IGF1R	Insulin-Like Growth Factor 1 Receptor
IGF1R	Insulin-Like Growth Factor Receptor Type 1
IGF2	Insulin-Like Growth Factor 2
IGF2R	Insulin-Like Growth Factor 2 Receptor
INS	Insulin
INSR	Insulin receptor
Insulin-R	Insulin receptor
IRS1	Insulin receptor substrate, type 1
IRS1	Insulin receptor substrate, type 1
IRS2	Insulin receptor substrate, type 2
IRS4	Insulin receptor substrate, type 4
JAGGED1	Jagged 1
mTOR	Mechanistic target of rapamycin
MTT	3-(4,5-dimethylthiazol-2-yl)-2,5-diphenyltetrazolium bromide
NOTCH1	Notch 1
P70S6K	p70 Ribosomal S6 kinase
PBS	Phosphate-buffered saline
PTEN	Phosphatase and tensin homolog
RPS6	Ribosomal Protein S6
SMI	Small molecule inhibitor
TBS	Tris-buffered saline
TSC2	Tuberous sclerosis complex 2
