# Supplementary material for: Chondrosarcoma: Multi-Targeting Therapeutic Effects of Doxorubicin, BEZ235, and the Small Molecule Aspartyl-Asparaginyl-β-hydroxylase Inhibitor SMI1182
Source: Cancers (Basel). 2025 May 15;17(10):1671. doi: 10.3390/cancers17101671 (PMC12109828; doi:10.3390/cancers17101671)
Supplement: Supplementary file 1 [file cancers-17-01671-s001.zip › cancers-3613133-supplement-high quality.pdf]

Supplementary Figures

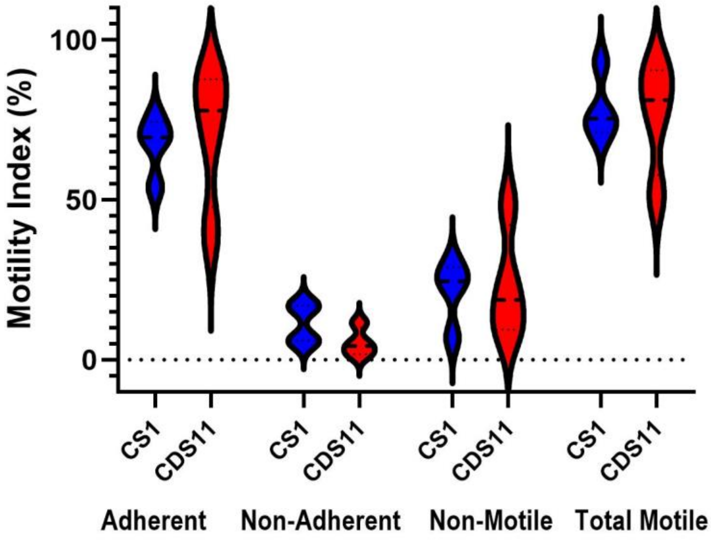

Figure S2. Directional Cell Motility and Adhesion in CS1 and CDS11 cells. These studies were performed using a Boyden chambertype apparatus and the ATPLite luminescence assay. The assay determined the percentages of non-motile (remaining on the upper membrane surface), motile adherent (migrated through the pores but still adherent to the undersurface of the membrane), motile non-adherent (migrated to the bo'om well of the chamber), and total motile (motile adherent+ motile non-adherent). Results were analyzed by two-way ANOVA. None of the differences were statistically significant.

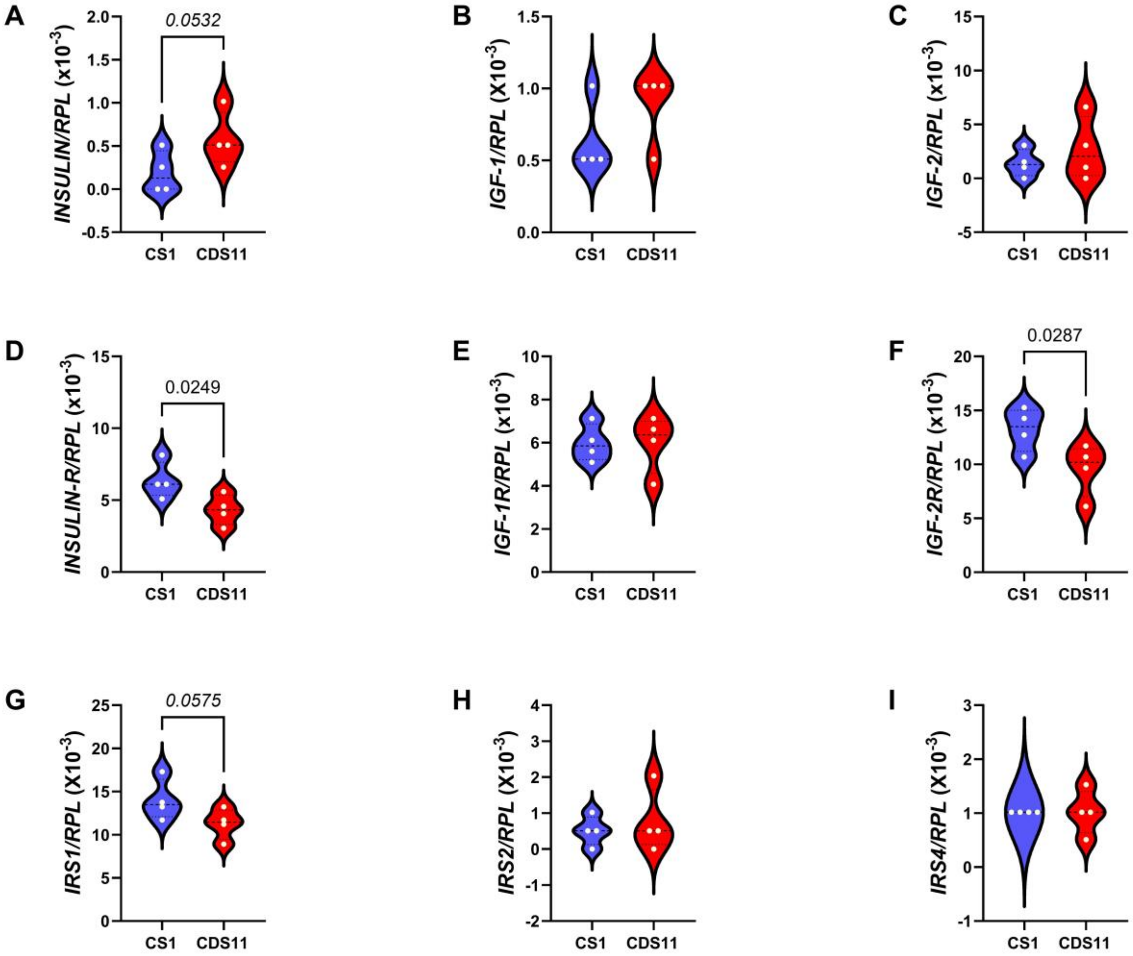

Figure S3. Insulin, IGF, and IRS Pathway Distinctions Between Grade 3 Versus Grade 2 Conventional Chondrosarcoma. CS1 (Grade 3) and CDS11 (Grade 2) cells were analyzed for (A) Insulin, (B) IGF-1, (C) IGF-2, (D) Insulin-R, (E) IGF-1R, (F) IGF-2R, (G) IRS-1, (H) IRS-2, and (I) IRS-4 mRNA levels were measured using a multiplex bead-based RNA hybridization protocol with results normalized to Ribosomal Protein L13a (RPL). Inter-group comparisons (n=4 cultures/group) were made by T-test analysis. Significant ($p \leqslant 0.05$) and statistical trendwise ($0.05 < p < 0.10$) (italics) p-values are displayed.

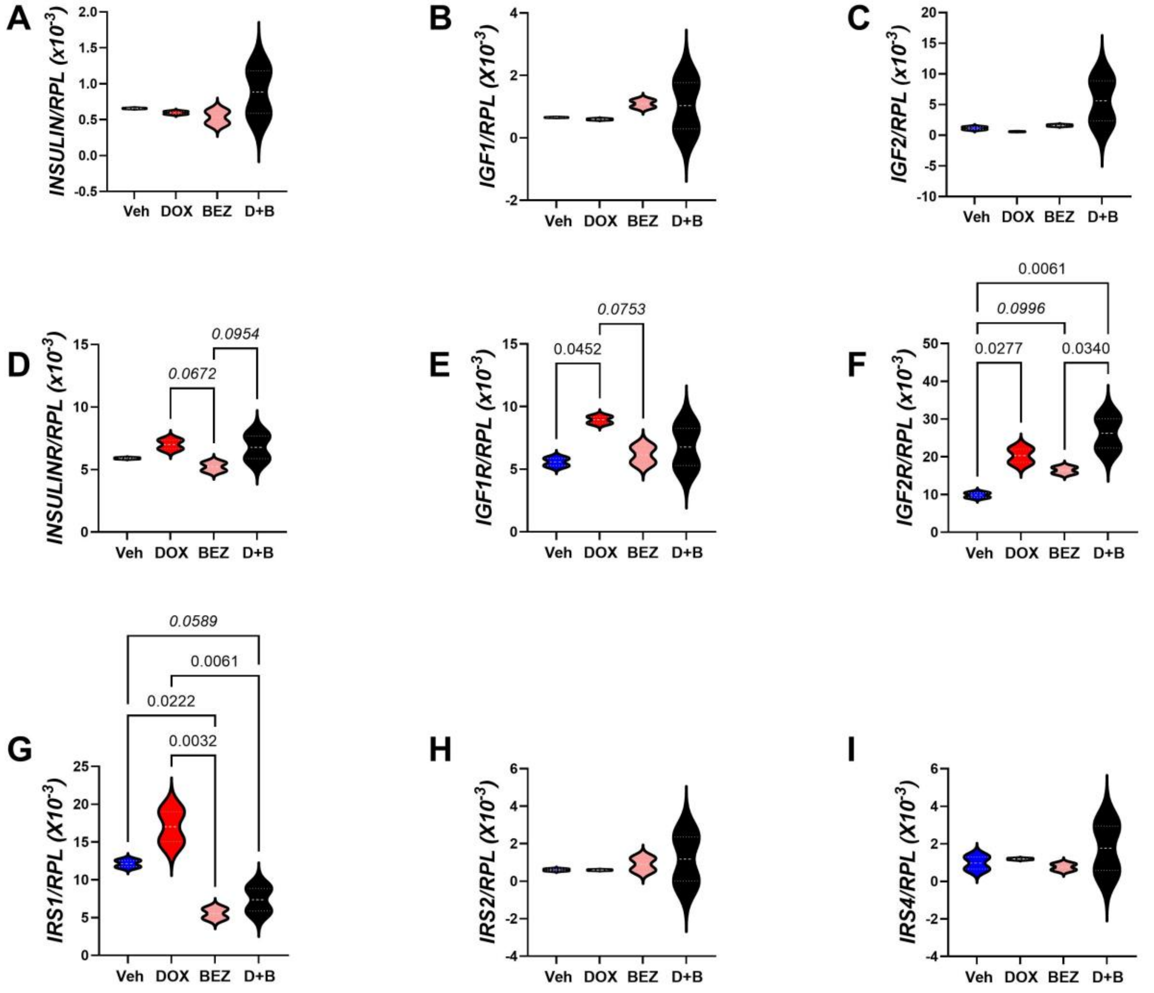

Figure S4. Molecular Pathways of DOX-, BEZ-, and DOX+BEZ (D+B)-Mediated CS1 Cytotoxicity Via Insulin, IGF, and IRS Pathways. CS1 cells were treated for 48 with Vehicle, DOX, BEZ, or D+B. (A) Insulin, (B) IGF-1, (C) IGF-2, (D) Insulin-R, (E) IGF-
1R, (F) IGF-2R, (G) IRS-1, (H) IRS-2, and (I) IRS-4 mRNA levels were measured using a multiplex bead-based RNA hybridization protocol with results normalized to Ribosomal Protein L13a (RPL). Inter-group comparisons were made by ANOVA (See Table 3) and the Tukey post hoc repeated measures test. Significant p-values are shown in the panels. Significant ($p \leqslant 0.05$) and statistical trendwise ($0.05<p<0.10$) (italics) p-values are displayed.

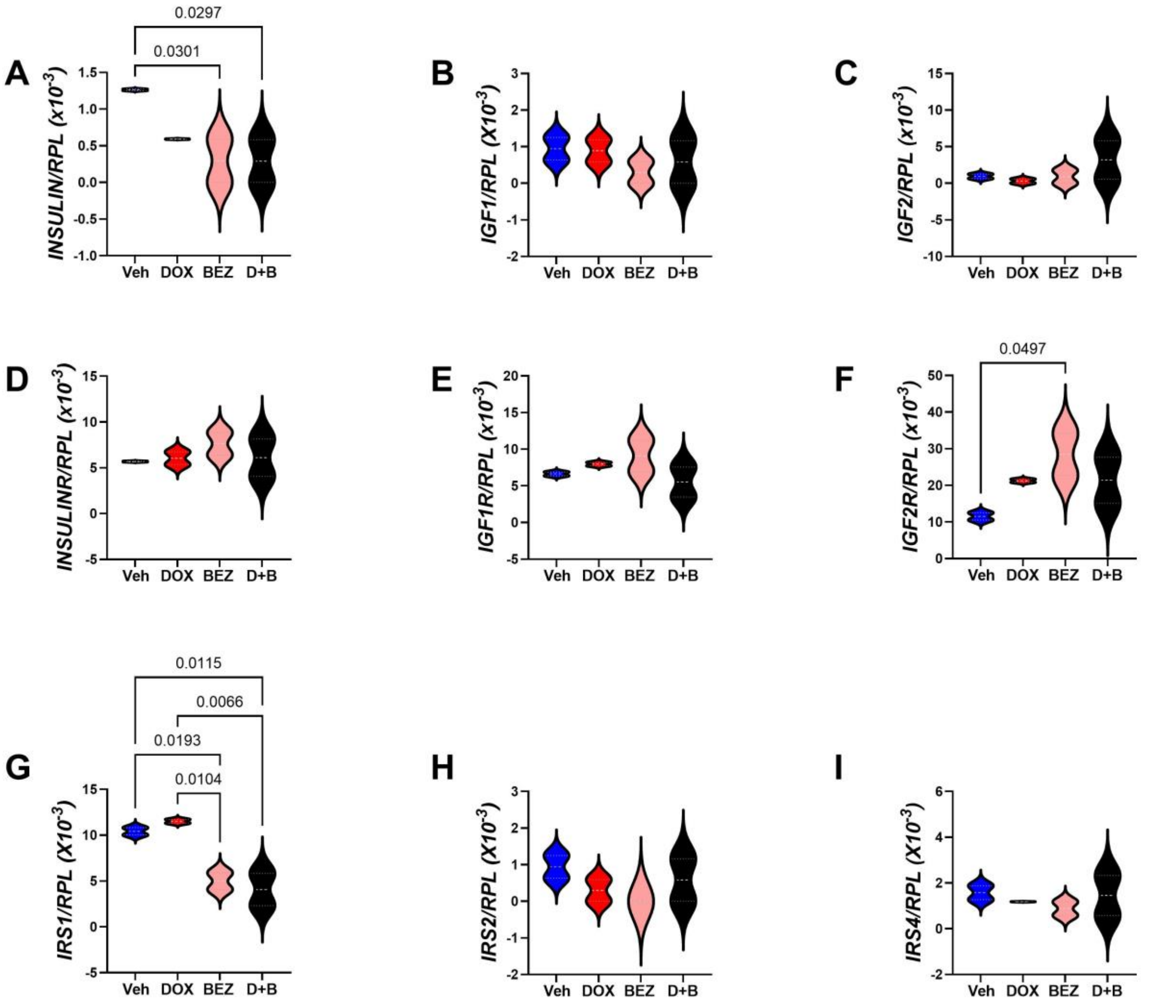

Figure S5. Molecular Pathways of DOX-, BEZ-, and DOX+BEZ (D+B)-Mediated CDS11 Cytotoxicity Via Insulin, IGF, and IRS
Pathways. CDS11 cells were treated for 48 with Vehicle, DOX, BEZ, or D+B. (A) Insulin, (B) IGF-1, (C) IGF-2, (D) Insulin-R, (E) IGF-
1R, (F) IGF-2R, (G) IRS-1, (H) IRS-2, and (I) IRS-4 mRNA levels were measured using a multiplex bead-based RNA hybridization protocol with results normalized to Ribosomal Protein L13a (RPL) (n=4 cultures per treatment group). Inter-group comparisons were made by ANOVA (See Table 4) and the Tukey post hoc repeated measures test. Significant ($p \leqslant 0.05$) and statistical trendwise ($0.05<p<0.10$) (italics) p-values are displayed.
