# Supplementary material for: Chondrosarcoma: Multi-Targeting Therapeutic Effects of Doxorubicin, BEZ235, and the Small Molecule Aspartyl-Asparaginyl-β-hydroxylase Inhibitor SMI1182
Source: Cancers (Basel). 2025 May 15;17(10):1671. doi: 10.3390/cancers17101671 (PMC12109828; doi:10.3390/cancers17101671)
Supplement: Supplementary file 1 [file cancers-17-01671-s001.zip › cancers-3613133-supplementary-high quality.pdf]

Figure S1: SMI1182 Synthesis Scheme

SMI-1182

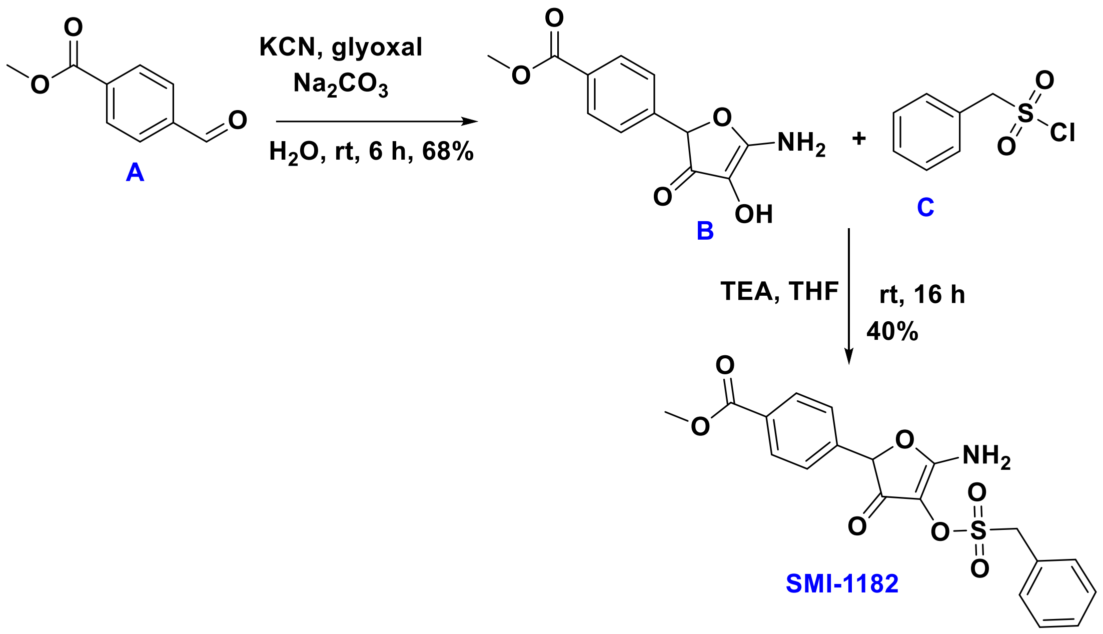

Step – 1:

Potassium cyanide (0.91 g) was added to sodium carbonate (1.7 g) in deionized water (30 mL) in a 3 – Neck Glass Round Flask and placed in an ice bath. The system was repeatedly purged using a vacuum pump and nitrogen gas. Glyoxal (3.72 g) was then added to the system without the introduction of O_2 and the reactants were allowed to dissolve with stirring. In a stoppered tube, the aldehyde (7.11 mmoles) was added to 1,4 - dioxane (5 mL) , purged , and then added drop - wise to the system . The system was then removed from the ice bath and allowed to stir at room temperature for 1 hour. After 1-hour, acetic acid (5 mL) was added drop - wise until gas bubbles were no longer visible from the addition of acetic acid, or until the solution was at a pH of less than 6. The solution was vacuum filtered and washed with ice cold water (5 mL) , methanol (5 mL) and ether (5 mL) and then was allowed to air dry . Crude material was recrystallized with methanol, collected by vacuum filtration and rinsed with diethyl ether and dried under vacuum.

Step – 2:

Compound **A** (1 eq) was dissolved in dry. THF and added TEA. The reaction mixture was cooled to 0^0C and then added Compound **B**. Reaction mixture was stirred for 30 mts at 0^0C and removed from the ice bath and stirred at RT for 16h. Progress of the reaction mixture was monitored by TLC. After the reaction completion, the formed precipitate was filtered and dried to obtain the title compound.

Methyl 4-(5-amino-4-((benzyl sulfonyl)oxy)-3-oxo-2,3-dihydrofuran-2-yl) benzoate (BT-1182):
Yield = 40%. ^{1}H NMR (400 MHz, DMSO) δ 8.82 (s, 2H), 8.05 (d, *J* = 7.9 Hz, 2H), 7.59 – 7.48 (m, 4H), 7.43 – 7.36 (m, 3H), 5.90 (s, 1H), 4.93 (q, *J* = 14.0 Hz, 2H), 3.88 (s, 3H); ^{13}C NMR (101 MHz, DMSO) δ 181.60, 173.93, 166.34, 140.35, 131.52, 130.38, 130.02, 129.15, 129.00, 128.95, 127.15, 107.78, 83.25, 57.75, 52.74.
